# Depleted carbon isotope compositions observed at Gale crater, Mars

**DOI:** 10.1073/pnas.2115651119

**Published:** 2022-01-18

**Authors:** Christopher H. House, Gregory M. Wong, Christopher R. Webster, Gregory J. Flesch, Heather B. Franz, Jennifer C. Stern, Alex Pavlov, Sushil K. Atreya, Jennifer L. Eigenbrode, Alexis Gilbert, Amy E. Hofmann, Maëva Millan, Andrew Steele, Daniel P. Glavin, Charles A. Malespin, Paul R. Mahaffy

**Affiliations:** ^a^Department of Geosciences, The Pennsylvania State University, University Park, PA 16802;; ^b^Earth and Environmental Systems Institute, The Pennsylvania State University, University Park, PA 16802;; ^c^NASA Jet Propulsion Laboratory, California Institute of Technology, Pasadena, CA 91109;; ^d^Solar System Exploration Division, NASA Goddard Space Flight Center, Greenbelt, MD 20771;; ^e^Climate and Space Sciences and Engineering, University of Michigan, Ann Arbor, MI 48109;; ^f^Department of Earth and Planetary Sciences and Earth-Life Science Institute, Tokyo Institute of Technology, Tokyo 152-8550, Japan;; ^g^Department of Biology, Georgetown University, Washington, DC 20057;; ^h^Earth and Planets Laboratory, Carnegie Institution for Science, Washington, DC 20015

**Keywords:** Gale crater, Mars, carbon isotopes, pyrolysis, methane

## Abstract

Obtaining carbon isotopic information for organic carbon from Martian sediments has long been a goal of planetary science, as it has the potential to elucidate the origin of such carbon and aspects of Martian carbon cycling. Carbon isotopic values (δ^13^C_VPDB_) of the methane released during pyrolysis of 24 powder samples at Gale crater, Mars, show a high degree of variation (−137 ± 8‰ to +22 ± 10‰) when measured by the tunable laser spectrometer portion of the Sample Analysis at Mars instrument suite during evolved gas analysis. Included in these data are 10 measured δ^13^C values less than −70‰ found for six different sampling locations, all potentially associated with a possible paleosurface. There are multiple plausible explanations for the anomalously depleted ^13^C observed in evolved methane, but no single explanation can be accepted without further research. Three possible explanations are the photolysis of biological methane released from the subsurface, photoreduction of atmospheric CO_2_, and deposition of cosmic dust during passage through a galactic molecular cloud. All three of these scenarios are unconventional, unlike processes common on Earth.

## Significance

Carbon isotopic analysis is among the most pervasive geochemical approaches because the fractionation of carbon isotopes produces a natural tracer of biological and chemical processes. Rover-based carbon isotopic analyses of sedimentary rocks on Mars have the potential to reveal modes of Martian carbon cycling. We report carbon isotopic values of the methane released during pyrolysis of samples obtained at Gale crater. The values show remarkable variation indicating different origins for the carbon evolved from different samples. Samples from multiple locations within Gale crater evolved methane with highly fractionated carbon isotopes. We suggest three routes by which highly fractionated carbon could be deposited on Mars, with each suggesting that Martian carbon cycling is quite distinct from that of the present Earth.

**Fig. 1.**
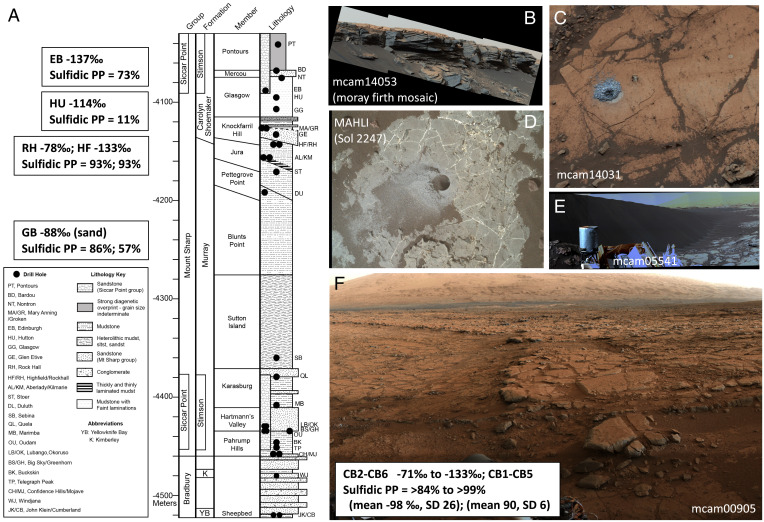
Geologic context of samples included in this study. (*A*) Stratigraphic column with labels for each of the MSL drill sites. (*B*) Moray_Firth Mastcam mosaic (mcam14053) from Sol 2685 showing Greenheugh pediment near the location of the EB drill hole, which was drilled on top. (*C*) HU drill hole in the Glasgow member of the Murray formation just below the Greenheugh pediment. (*D*) HF drill hole in gray-colored Jura member Murray mudstone at the top of the VRR. (*E*) Namib dune of the Bagnold dunes where the GB sample was taken. (*F*) Yellowknife Bay locality where the CB drill hole was drilled into mudstone of the Sheepbed member of the Bradbury group rocks.

Carbon isotopic data from Martian sedimentary organic carbon can potentially elucidate the origin of indigenous organics and reveal aspects of the Martian carbon cycle. Extended exploration by the Mars Science Laboratory (MSL) *Curiosity* rover of the fluvio-lacustrine sedimentary system at Gale crater provides unique opportunities, as samples are collected from a variety of locations within a known stratigraphic context (1, 2). MSL has collected and analyzed more than 30 drilled samples on Mars between August 2012 and July 2021. These samples have been collected from varied lithologies from hundreds of meters of stratigraphy in Gale crater that represent the complex history and evolution of the region. A description of the mission samples studied can be found in the supplement, and a stratigraphic column is shown in Fig. 1*A*. Methods used in this work are also described in the supplement to this manuscript. This study considered the carbon isotopic values of methane evolved during pyrolysis as observed by the MSL tunable laser spectrometer (TLS) of the Sample Analysis at Mars (SAM) instrument suite (3) from 24 samples from Gale crater, Mars. Methane abundances and δ^13^C values were determined from analyzing TLS-SAM high-resolution spectra.

## Results

For evolved gas analysis (EGA) at Gale crater, drilled powder or scooped fines are heated at 35 °C min^−1^ under a helium flow in quartz cups up to ∼850 °C. A fraction of the evolved gas mixture is directly analyzed with a quadrupole mass spectrometer, and a specific preselected temperature cut of the remaining gas is diverted to the Herriott cell of the TLS. The redox state of the oven, in practice, depends on the relative abundances of oxychlorine species and reduced minerals in each sample. During EGA, organic material can produce a range of products including CO_2_, CH_4_, CO, OCS, CS_2_, and molecular organic fragments. EGA δ^13^C CH_4_ values were measured by the TLS instrument of the SAM suite for 24 samples from Gale crater, Mars (Table 1) using methods described in this paper’s supplement. The amount of CH_4_ observed by the TLS-SAM instrument for the TLS temperature cut is also indicated in Table 1. This includes five analyses of the Cumberland (CB) sample drilled in the Sheepbed member of the Bradbury group rocks at Yellowknife Bay, as well as 15 samples from the Mount Sharp group, three from the overlying Stimson formation, and one scooped sand sample. Shown in Table 2 are the posterior probabilities of reduced sulfur presence (based on several evolved sulfur gases observed compared to laboratory data) and the δ^34^S values calculated from SO_2_ evolved between ∼500 and 600 °C during EGA and measured by the SAM quadrupole mass spectrometer (QMS).

**Table 1.** MSL methane isotopic values from EGA

Label*	Sol	Temperature cut for TLS (°C)	TLS CH_4_ (nmol)	±1 SE	TLS CH_4_ δ^13^C (‰)	±1 SE
CB1	281	220–319	5.0	<0.01	−133	12
CB2	286	99–349	5.5	0.01	−115	5
CB3	290	450–786	2.6	0.01	−96	8
CB5	368	450–786	1.8	<0.01	−75	9
CB6	382	450–786	1.3	0.01	−71	21
TP	928	382–614	6.9	0.01	−30	12
BS1	1,130	639–862	12.1	0.01	−32	9
GH1	1,147	639–862	7.7	0.01	11	16
GB2	1,237	583–770	6.2	0.01	−88	8
OU1	1,382	347–749	43.8	0.03	−17	2
MB	1,443	377–771	19.1	0.06	−1	6
DU	2,072	539–837	27.2	0.06	−45	3
ST	2,147	437–788	45.9	0.06	−31	2
HF	2,231	167–356	16.3	0.02	−133	4
RH	2,281	405–572	2.1	0.00	−78	7
KM	2,393	374–549	14.4	0.02	−35	6
GE1	2,497	350–600	39.8	0.03	−57	2
GE3	2,531	355–617	19.3	0.04	−37	5
HU	2,676	452–765	20.1	0.02	−114	3
EB	2,721	264–538	8.4	0.02	−137	8
GG	2,765	270–556	77.9	0.68	22	10
MA1	2,844	270–556	34.7	0.04	−29	2
BD	3,098	220–440	38.9	0.05	−28	2
PT	3,176	220–440	27.7	0.03	−65	2

^*^Labels refer to *Curiosity* rover drill holes: Cumberland Cumberland (CB1, CB2, CB3, CB5, CB6), Telegraph Peak (TP), Big Sky (BS1), Greenhorn (GH1), Gobabeb (GB2), Oudam (OU1), Marimba (MB), Duluth (DU), Stoer (ST), Highfield (HF), Rockhall (RH), Kilmarie (KM), Glen Etive (GE1, GE3), Hutton (HU), Edinburgh (EB), Glasgow (GG), Mary Anning (MA1), Bardou (BD), and Pontours (PT).

SE = SE at 67% CI.

**Table 2.** Relevant results from the MSL QMS during EGA and informal stratigraphic units for samples

Label	BSW (nmol)	Reduced S PP* (%)	SO_2_ δ^34^S† (‰)	±1 SE	Stratigraphy
CB1	3	>99	−21	36	Sheepbed, Bradbury group
CB2	1.8	89	−28	14	
CB3	1.3	89	−47	14	
CB5	0.2	84	−40	10	
CB6	0.1	ND	ND	ND	
TP	0.2	<1	6	7	Pahrump Hills‡
BS1	0.4	78	3	6	Stimson formation
GH1	0.2	<1, 60	28	7	
GB2	0.1	86, 57	ND	ND	Bagnold dunes
OU1	0.6	16	−31	9	Hartmann's‡
MB	0.2	74	ND	ND	Karasburg‡
DU	31.2	2	ND	ND	Blunts Point‡
ST	2.9	4	ND	ND	Pettegrove Point‡
HF	3.5	93	ND	ND	Jura‡ (including VRR for HF and RH)
RH	0.3	93	−18	40	
KM	0.4	97	−21	19	
GE1	0.6	7	20	4	Knockfarril Hill§
GE3	0.2	9	−14	5	
HU	12.7	11	18	6	GG§
EB	5	73	−27	7	Stimson formation
GG	1.2	1	5	9	GG§
MA1	0.3	1	8	5	Knockfarril Hill§
BD	0.5	6	−9	6	Mercou§
PT	0.3	1	1	3	Pontours§

ND, not determined.

* PP, posterior probability; Wong (5); Wong et al. (6).

^†^Franz et al. (4); Wong (5).

^‡^Members of Murray formation.

^§^Members of Carolyn Shoemaker formation.

**Fig. 2.**
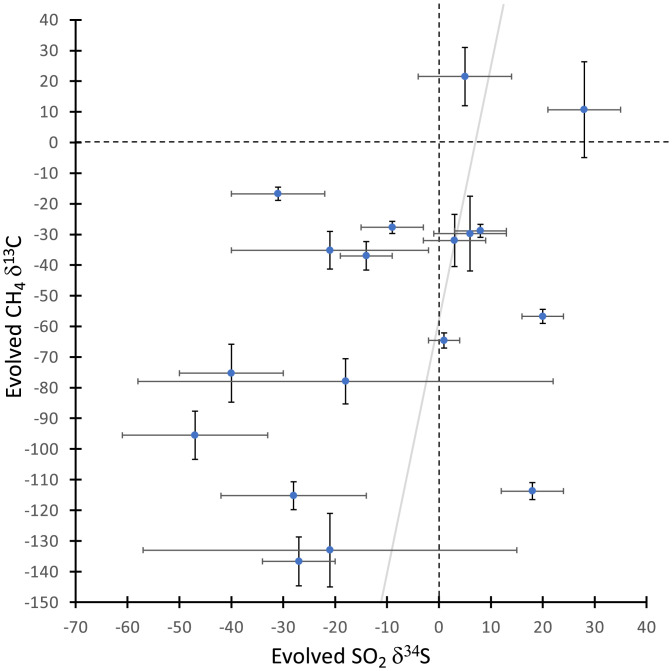
EGA TLS CH_4_ δ^13^C_VPDB_ values versus EGA SO_2_ δ^34^S_VCDT_ QMS values (4, 5). Error bars indicate 1 SE. For reference, the dashed lines separate the graph into quadrants around the origin, and the gray line shows a weighted linear fit (*y* = [8 ± 3]*x* − [59 ± 12], mean squared weighted deviation = 7). Most analyses from Gale crater that have a large negative δ^13^C value in evolved CH_4_ also have ^34^S-depleted evolved SO_2_.

As seen in Fig. 2, the TLS CH_4_ δ^13^C values that are highly ^13^C depleted correspond predominantly with the ^34^S-depleted δ^34^S QMS values observed for evolved SO_2_, reported by Franz et al. (4) and Wong (5). Four of the most depleted TLS CH_4_ δ^13^C values (Table 1) are from samples [CB2, CB3, CB5, and Edinburgh (EB)] that also clearly evolve ^34^S-depleted SO_2_ (Table 2 and Fig. 2). Such δ^34^S-depleted SO_2_ evolved at mid (500 to 600 °C) temperature has been interpreted to indicate Martian sulfides (4). Reduced sulfur has also been inferred based on the evolution of OCS and CS_2_ compared to analyses of laboratory sulfur samples (6). Interestingly, 8 out of the 10 samples showing strong TLS δ^13^C depletions (i.e., <−70‰) in evolved CH_4_ also evolved sulfur gases (OCS and CS_2_) indicative of a reduced sulfur detection based on statistical comparisons to laboratory runs (Table 2). One of the two samples in which the EGA data do not support a reduced sulfur detection is CB6, which had an unusual EGA procedure that prohibited an effective analysis of the sulfur gases. The other is Hutton (HU), which occurs just below the Greenheugh pediment (a gently sloping erosional surface downslope from Gediz Vallis) and the Basal Siccar Point group unconformity. HU shows evidence for geochemical alteration by later fluids flowing near the Basal Siccar Point group unconformity (e.g., ref. 7).

## Discussion

Some of the ^13^C depletions reported here are anomalously large, especially with respect to the carbon isotopic composition of the Martian atmosphere, whose δ^13^C value reported earlier by TLS-SAM is ∼+46‰ (8). This atmospheric value reflects the integrated largescale loss of volatiles from the Martian atmosphere and thus, the δ^13^C composition of the atmosphere may have been less ^13^C enriched when Gale crater sediments were deposited. Because of the magnitude of the ^13^C depletions observed in CH_4_ evolved during EGA runs, the authors have considered potential rover-induced origins for the observations without uncovering any explanation. In fact, the TLS spectra obtained on Mars from evolved CH_4_ (*SI Appendix*, Fig. S1) are exceptionally clean and provide multiple ^12^C and ^13^C lines with which to calculate δ^13^C values, making it unlikely that the ^13^C depletions observed are due to an interfering organic molecule. From the repeat CB analyses, it appears that the isotope depletion observed in CH_4_ is most pronounced at lower temperatures. This observation suggests that precursors to the evolved CH_4_ are relatively volatile organic molecules. However, strong depletions were still observed in several samples using high-temperature cuts (>450 °C) in which the temperature cut includes a tail of a CH_4_ release centered at a lower temperature (e.g., *SI Appendix*, Fig. S2). The CH_4_ isotopic variation in CB samples, though, are not completely explained by differences in the temperature cut. Additionally, at Yellowknife Bay, the TLS analyses of CB using the 2.78-μm laser produced highly ^13^C-depleted CO_2_ δ^13^C values (*SI Appendix*, Table S1). While these TLS analyses of CB produced CO_2_ δ^13^C values that were, in some cases, comparable to CH_4_ δ^13^C (*SI Appendix*, Table S1), such depleted CO_2_ δ^13^C values have not been observed in later samples of the mission. In contrast, TLS CH_4_ δ^13^C values using the 3.27 μm laser show strong depletions in multiple different drill samples from vastly different parts of the mission. Because the observation of anomalous ^13^C values in evolved CH_4_ are repeatable with different samples spread out in space and time, we have focused on those values for this study.

We have considered the SAM instrument background of *N*-tert-butyldimethylsilyl-*N*-methyl-trifluoroacetamide (MTBSTFA (δ^13^C = −35‰; refs. 9 and 10) as a reasonable source of evolved CH_4_ to consider. Early in the mission, this background was estimated to contribute up to 900 nmol of CO_2_ during pyrolysis (11). In addition to oxidizing to CO_2_, MTBSTFA is known to react with water resulting in 1,3-bis(1,1-dimethylethyl)-1,1,3,3-tetramethyldisiloxane [or bisilylated water (BSW)], which can be monitored by the SAM QMS. Based on the levels of BSW detected, the level of MTBSTFA background has been variable between runs depending on how long it has been since a wet chemistry experiment and, for a few cases in which it was relevant, how long a sample was stored before analysis. For 16 of the samples, the mean of the total BSW observed during the course of the EGA runs was 1.0 nmol with an SD of 1.5 nmol (Table 2). HU had over 22 times this average, and Duluth (DU) had over 30 times more than this average. In the case of DU, the extreme amount of BSW observed was also concurrent with an anomalous amount of total EGA methane (774 nmol) and a TLS CH_4_ isotopic value (δ^13^C = −45‰) similar to MTBSTFA (δ^13^C = −35‰), which demonstrated that with elevated levels of MTBSTFA in the SAM background, a portion can end up as evolved CH_4_. Further, an intramolecular isotopic analysis of methyl-trifluoroacetamide from the hydrolysis of MTBSTFA showed that the types of carbon (methyl-carbon and carbonyl C) most likely to contribute CH_4_ from the MTBSTFA background were the most ^13^C enriched (*SI Appendix*, Fig. S3), making it unlikely that the observed anomalies stemmed from a site-specific carbon depletion obscured in the bulk δ^13^C value for MTBSTFA.

Major endmember carbon reservoirs (*SI Appendix*, Table S4) presently on Mars are the atmospheric CO_2_ (δ^13^C = +46 ± 4‰; ref. 8) and the igneous carbon (δ^13^C = −20 ± 4‰; ref. 12). TLS δ^13^C values between −17 ± 2‰ and −57 ± 2‰ were found for 10 samples including DU. The isotopic composition of the CH_4_ evolved during pyrolysis of these samples may reflect the MTBSTFA SAM background, Martian igneous carbon, and/or meteoritic infall along with any isotopic fractionations the occur during oven reactions. Laboratory experiments using solid materials were conducted to explore the magnitude of carbon isotopic fractionation possible during pyrolysis under conditions similar to SAM (*SI Appendix*, Table S2). We found that cleavage and reduction of methyl groups to CH_4_, as would happen for most CH_4_ derived from MTBSTFA products, produced little ^13^C depletion (0.4 to 4.6‰; *SI Appendix*, Fig. S4). CH_4_ evolved from recalcitrant sources (graphite and diamond) showed ^13^C-depletion of 8 to 21‰, CH_4_ from oxalate/oxamide showed moderate ^13^C depletion (25.0 to 25.3‰), and bicarbonate reduction showed the largest ^13^C depletions (28.5 to 49‰). Both our laboratory pyrolysis experiments (*SI Appendix*, Table S2) and modeling of possible isotopic pseudoequilibration during pyrolysis (*SI Appendix*, Figs. S5–S8 and Table S3) considering several different scenarios suggested that oven processes would typically produce fractionations less than 50‰ and, therefore, cannot account for the large ^13^C depletions observed in multiple samples at Gale crater. Applying the most extreme oven fractionation imagined to these carbon reservoirs results in CH_4_ with δ^13^C values of about −5 to −70‰. CB (CB1, CB2, CB3, CB5, CB6), Gobabeb (GB2), Highfield (HF), Rock Hall (RH), Hutton (HU), and EB showed TLS CH_4_ values more ^13^C depleted than this range, indicating that oven reactions are not likely to be causing their anomalous ^13^C-depleted values observed in evolved CH_4_.

It may be notable that the highly depleted ^13^C values for evolved CH_4_ have so far been found in five distinct locations at Gale crater, Mars (Table 2 and Fig. 1 *A–F*). The highly ^13^C-depleted signal was first seen in the mudstones of Yellowknife Bay on the crater floor (Fig. 1*F*) in a location where high thermal inertia values were measured from orbit and have been potentially attributed to secondary alteration at the end of the Peace Vallis fan (13). Next, highly ^13^C-depleted methane values were observed in a sample of the sand from the Bagnold dunes (GB2), a modern dune field of basaltic sand (Fig. 1*E*). Next, the depleted values were observed at the top of the Vera Rubin ridge (VRR) in mudstone samples of the red and gray Jura member of the Murray formation (RH and HF, respectively). Finally, the highly ^13^C-depleted values were observed just below the Basal Siccar Point group unconformity (HU; Fig. 1*C*) and in the overlying Stimson formation sandstone that forms the cap rock of the current Greenheugh pediment morphological feature (EB; Fig. 1*B*). These different locations include a variety of lithologies (mudstone, sand, and sandstone) and are temporally spread throughout the mission operations to date.

**Fig. 3.**
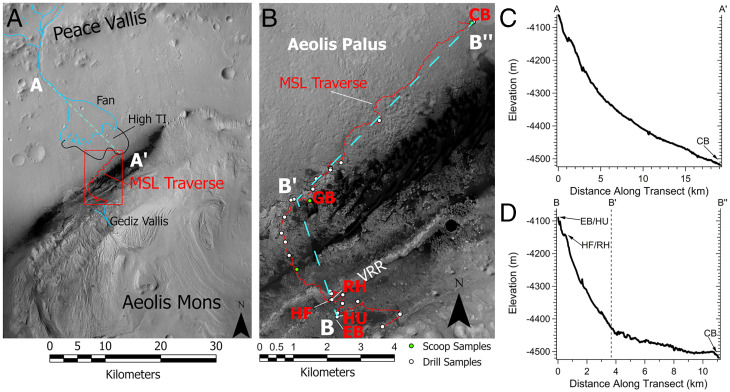
(*A*) Map of the northwest portion of Gale crater with annotations showing Peace Vallis and the alluvial fan leading toward the high thermal inertia region (High TI) in Aeolis Palus. Gediz Vallis is labeled to the south of the MSL traverse. The MSL traverse through sol 3192 is shown in red. The red rectangle outlines the region shown in *B*. Dashed line represents the profile in *C*. Base map is a mosaic from Calef and Parker (84). (*B*) Map of the MSL-specific study area and rover traverse through sol 3192. Samples analyzed by TLS with highly depleted ^13^C values are labeled along the traverse. Dashed line corresponds to the elevation profile shown in *D*. Base map is a HiRISE mosaic from the Planetary Data System (PDS) PLACES archive. (*C*) Profile from A to A′ in *A* showing the change in elevation from the lower end of Peace Vallis to Yellowknife Bay. (*D*) Elevation profile from B to B′ to B″ shown in *B*. Drill samples with highly depleted ^13^C values are labeled with approximate elevations.

**Fig. 4.**
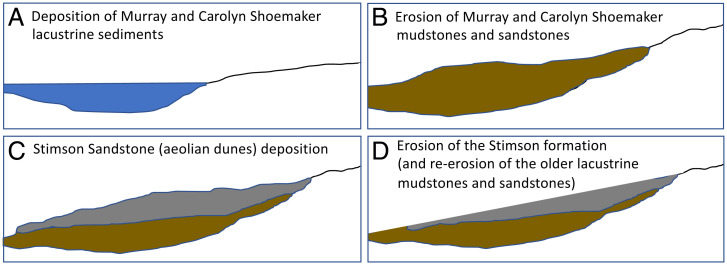
Notional geologic history for Gale crater progressing from left to right starting with (*A*) a lacustrine environment. (*B*) After deposition, the lacustrine mudstones and sandstones were later exposed and eroded to produce the unconformity between the Mount Sharp group rocks (Murray and Carolyn Shoemaker formations) and the Stimson formation. (*C* and *D*) The Stimson sandstone has also been eroded to produce a paleosurface (and later the present landscape of ridges, buttes, and a pediment).

One potential connection between these evolved methane samples depleted in ^13^C is that they might all represent samples associated with a paleosurface (Figs. 3 and 4). The locations of the samples yielding depleted ^13^C methane along with their elevations are shown in Fig. 3 for illustration of this paleosurface possibility. Yellowknife Bay may have been weathered by flow out of Peace Vallis. The Bagnold dunes represent sand from, in part, a recently eroded local sandstone. The VRR is a resistant topographic ridge relatively higher than other samples of the Murray formation mudstone with a unique diagenetic history (14) that may include weathering from the outflow of the Gediz Vallis (6). Finally, the Greenheugh pediment is presently a large geomorphic surface and represents the remains of a larger paleosurface that would have extended downslope toward the crater floor (15). The HU sample, which shows the strongly ^13^C-depleted signature, is just below the Greenheugh pediment and is believed to have been exposed to fluid migration near the Basal Siccar Point group unconformity (7). Similar to the HU sample, Pontours (PT) is in the Carolyn Shoemaker formation below the Greenheugh pediment and, therefore, has likely been exposed to fluids during erosion of the pediment. PT is actually at a higher elevation than the EB sample because of the lateral slope of the pediment, and, interestingly, the TLS CH_4_ δ^13^C value for PT is intermediate between what is expected for SAM’s MTBSTFA background carbon and the highly ^13^C-depleted values (i.e., <−70‰) found for 10 of the Gale crater samples.

While this distribution of samples yielding a strongly ^13^C-depleted signature may be a coincidence, it could be explained if the signature relates to a process that primarily impacted a paleosurface associated with outflow from Gediz Vallis and Peace Vallis that included the Greenheugh pediment capping unit, the topographic high represented by the VRR, and the crater floor at Yellowknife Bay. Given this distribution of samples yielding isotopically anomalous methane, we have considered multiple phenomena that have the potential to explain the results. The phenomena discussed are not presented in order of preference, but rather are treated equally, starting with the canonical explanation for when this type of geochemical signature is found on Earth and moving to possible processes that would be increasingly specific to Mars.

### Martian Methanotrophy?

The observed ^13^C-depleted EGA CH_4_ was presumably released from organic materials in the solid sample during pyrolysis. The canonical explanation on Earth for highly depleted carbon associated with a paleosurface would be microbial methanotrophy converting methane into biomass when the methane was already ^13^C depleted because of its biological production during methanogenesis (e.g., ref. 16). The Archean Tumbiana Formation has highly ^13^C-depleted biomass preserved along with abundant lacustrine stromatolites (e.g., refs. 17 and 18). The bulk biomass found in the Tumbiana Formation has δ^13^C values as low as −60‰ (19), the origin of which is often attributed to widespread methanotrophy during a period of abundant methane in the Earth’s atmosphere and shallow basins (16, 20). Highly depleted biomass is also found in some sediments in and around active modern marine methane seeps (e.g., refs. 21–24), and there are several examples of highly ^13^C-depleted biomass preserved in paleo seeps deposits (25, 26).

Because of both the overall oxidizing state of the Martian atmosphere and the presence of ferric iron and sulfate minerals, during times of large methane releases, methanotrophy would be energetically favorable. Based on observations of apparent methane plumes from the Martian interior and the growing understanding of the ways methane can be microbially oxidized on Earth, microbial methanotrophy has been proposed as a possible metabolism for recent and ancient Mars (27, 28). This explanation would also potentially explain the observation of reduced sulfur in many of the same samples in which negative δ^13^C values were found (29) because marine methane oxidation is coupled to sulfate reduction. If the highly ^13^C-depleted carbon and reduced sulfur are indeed found predominantly together on a paleosurface, the co-occurrence could result from the coproduction of ^13^C-depleted microbial biomass and sulfides during the microbial anaerobic oxidation of methane based on sulfate as an electron acceptor.

In order to get the observed magnitude of ^13^C depletion in the observed EGA methane released from organic materials, the CH_4_ that was originally consumed by microorganisms in this model would need to already be highly ^13^C depleted (perhaps −40 to −100‰) because the observed depletion on Earth during anaerobic methanotrophy is on the order of about 30‰ (30), assuming fractionations associated with possible Martian metabolisms are similar to those observed on Earth. Therefore, this explanation requires multiple steps with the CH_4_ consumed by the methanotrophs to be biological CH_4_ from microbial methanogenesis. Furthermore, the inorganic CO_2_ fueling this microbial ecosystem would need to have a δ^13^C composition similar to Martian magmatic carbon (−20‰; refs. 12 and 31) rather than the highly ^13^C-enriched values observed in the present Martian atmosphere (+46‰; ref. 8).

For this model to work, either the deposition of the ^13^C-depleted carbon would have needed to occur before the loss of significant carbon from the Martian atmosphere or there would need to have been CO_2_ reservoirs in the Martian subsurface that were isolated from the atmosphere. In either scenario, the initial carbon could have had an isotopic composition down to −20‰. The first option, however, appears inconsistent with Gale crater sedimentology, given the relatively late emplacement of the Stimson formation that is cut by the paleosurface of interest here. The other option is possible, but it is hard to evaluate with our limited knowledge of the hydrodynamics of subsurface Mars. Potentially supporting this model is the observation of long, straight alkanes in the CB sample (32). Overall, there is, however, no supporting sedimentological evidence for microbial methanotrophy on the paleosurface discussed here. This is in sharp contrast to analog methanotrophic environments on Earth, for which there is often plenty of textural evidence for microbial processes in deposited layers. It is plausible that such evidence once existed at Gale crater as an authigenic carbonate (e.g., refs. 33 and 34) that was later dissolved by acidic fluids. While sedimentary structures may be destroyed by dissolution, dissolution can concentrate molecular biosignatures. The *Curiosity* rover will again encounter the Stimson formation sandstone on the Greenheugh pediment, providing an opportunity to search for specific organic molecules (e.g., refs. 35 and 36) in this rock unit. The lack of sedimentary evidence for microbial surface activity and the need to avoid influence from a ^13^C-enriched Martian atmosphere cast enough doubt on this biological explanation of surface methanotrophy to tentatively dismiss it pending further exploration for either evidence of surface-associated microbial activity or microbial-influenced sediments whose organic material could have been redeposited to the paleosurface.

### Interstellar Dust?

The solar system passes through an average-sized giant molecular cloud (GMC) once every 100 million years, providing a mechanism for triggering cooling events on terrestrial planets through the influx of particles to planetary atmospheres that is substantially higher (20 to 100 times) than the ongoing flux of interplanetary dust (37). The solar system passing through a dense molecular cloud would inevitably also result in an influx of ^13^C-depleted carbonaceous particles (38), as about 1% of such interstellar clouds is dust (39). The δ^13^C value for interstellar dust in the Allende meteorite has been shown to be as low as −260‰ (40), demonstrating that interstellar dust is a potential source of highly depleted carbon for periodic deposition on the surface of Mars. It is also plausible, but presently uncertain, that these particles would similarly be associated with sulfides having negative δ^34^S values, as observed in the *Curiosity* data. The flux of such particles would be quite low and typically diluted by other sources of carbon. However, because the arrival of such particles is predicted to trigger a global cooling event, it is reasonable that particles would accumulate on the surface of glacial ice largely absent of typical sedimentary carbon. Glacial melt during the glacial period and ice retreat after should leave the interstellar dust particles on the glacial geomorphological surface. The existing sedimentological evidence for the paleosurface that has cut the Stimson formation does not rule out glacial processes, with *Curiosity* having only visited the Greenheugh pediment surface briefly at EB, and some studies across Mars support such an interpretation for similar outflow channels (41–44).

This explanation of interstellar dust as a source of the observed ^13^C-depleted values at Gale crater largely fits the observations if the isotopic signatures are not diluted by other geological processes and with the understanding that this explanation relies on a rare event, making it perhaps coincidental that the mission should find this signature preserved. Finally, the SAM results presented here suggest that the most ^13^C-depleted values are liberated during relatively low oven temperatures, with the high-temperature examples typically being tails of lower-temperature methane release. These observations might be understood to indicate that the organic precursor molecules being converted into methane in the SAM oven are not recalcitrant kerogen-like molecules, as pyrolysis of kerogen usually occurs at a higher temperature. If this interpretation is correct, this constraint can be consistent with an interstellar dust origin, as there are a wide variety of known interstellar molecules, including many that are highly volatile (45). Overall, this explanation is plausible, but it requires additional research outside the scope of this report to verify, including the δ^34^S values and the nature and relative amounts of carbon forms in GMC dust.

### Abiotic Reduction of CO_2_?

There is growing recognition of the possible abiotic production of organic carbon on Mars through either electrochemical reduction (46) or photochemical reduction (10). In both cases, CO_2_ would be converted into organic material abiotically and therefore could, in principle, lead to ^13^C-depleted organic material associated with a paleosurface. For electrochemical reduction, sulfides are one of the various reduced minerals that thermodynamically drive such carbon fixation reactions, and, similarly, sulfides are possible mineral catalysts that can promote photochemical reduction of CO_2_. Thus, if abiotic reduction of CO_2_ is an important process on Mars, it is likely to be associated with reduced sulfur, as observed at Gale crater. The present atmosphere of Mars, as previously noted, is highly enriched in ^13^C (+46‰), and so either the abiotic reduction would need to have been occurring in subsurface environments (which does not match our observations) or would need to have occurred prior to the loss of much of the Martian atmosphere (which appears somewhat inconsistent with the timing of the erosional paleosurface with which the depleted carbon appears to be associated). While the timing of the erosional paleosurface is poorly constrained to the Hesperian, deuterium enrichment of clays at Gale crater indicated significant loss of atmospheric volatiles by this time (47), and the implications of that loss for the overall history of Martian volatile loss is an area of active research (48). Overall, though, the carbon isotopic fractionations expected during abiotic reduction are likely similar to those measured for abiotic hydrothermal reduction (up to ∼50‰; *SI Appendix*, Fig. S9*B*). Fractionation of this magnitude would not produce organic material as depleted as the observed TLS values for the EGA methane reported in Table 1, and therefore, this explanation does not appear to explain the observations of highly ^13^C-depleted evolved methane during pyrolysis of samples at Gale crater. However, carbon isotopic fractionation during photochemical reduction of CO_2_ on a mineral surface has not been experimentally evaluated, and so, further research is needed before we can fully evaluate the abiotic reduction as the source of the highly ^13^C-depleted carbon isotopic values reported here. To be clear, there are a number of other CH_4_ isotope values reported in Table 1 that could reflect the reduction of CO_2_ on the Martian surface, including the most ^13^C-enriched values (−1 ± 6‰ to +22 ± 10‰). These ^13^C-enriched results match what would be expected for processes, biotic or abiotic, that produce organic material from the modern Martian atmosphere, for example.

### Photolysis of CH_4_ or CO and SO_2_?

Photochemical reactions are attractive as a source for the observed phenomenon because photochemical reactions are known to have influenced Mars’s volatiles, including the sulfur isotopic values found in Martian meteorites (49–52). Also, the fallout of products from atmospheric photochemistry might be expected to accumulate on an erosional surface, such as the proposed paleosurface discussed throughout this paper. There are several different overall schemes by which photochemistry in the Martian atmosphere might result in the large δ^13^C depletions observed by the MSL-TLS component of the SAM instrument.

A growing number of studies have reported variable CH_4_ abundances in the Martian atmosphere (53–55), including recent reports of seasonal and diurnal variation (56, 57) and plumes (55) detectable on the surface at Gale crater. The photolysis of atmospheric CH_4_ in a relatively dry, anaerobic atmosphere can produce organic aerosols and particles (e.g., Ref. 58). Perhaps when the postulated paleosurface was exposed, CH_4_ photolysis resulted in the deposition of such organic material. This model would explain why the highly depleted δ^13^C values seem to be associated with a possible paleosurface, similar to a model proposed for the Late-Archean Earth (59). Further, the model could potentially also explain the observation of ^34^S-depleted reduced sulfur in the same deposits if there was also SO_2_ photolysis along with the CH_4_ photochemistry producing sulfur-containing organic molecules. For example, Halevy (60) suggested that photoexcitation of SO_2_ can lead to the production of methanesulfonic acid on the Archean Earth. Additional work is needed to explore the possibility that CH_4_ photolysis during Martian plume events would deposit ^34^S-depleted sulfur-containing organic material. However, past studies indicate that the magnitude of carbon isotopic fractionation during CH_4_ photolysis is relatively small (<15‰; refs. 61–63). Fractionation during CH_4_ photolysis appears insufficient to produce δ^13^C values as depleted as some of those observed at Gale crater even when starting from bulk Mars δ^13^C values (−20‰) representative of carbon from the interior of Mars. In order to produce organic material that is highly ^13^C depleted (considerably more depleted than −70‰), another step with significant δ^13^C fractionation would need to have occurred during the formation of the methane. In principle, this could be abiotic CO_2_ reduction in the Martian crust during serpentinization or microbial methanogenesis. As noted previously, the abiotic reduction of CO_2_ results in ∼50‰ ^13^C depletion. If the source CO_2_ had a δ^13^C composition of −20‰, subsequent abiotic reduction to CH_4_, and subsequent photochemistry, then the resultant organic material deposited could be as depleted as perhaps −85‰, a value that does not adequately include all of the values observed at Gale crater. If microbial methanogenesis were producing the observed atmospheric CH_4_, photolysis of the biologically produced CH_4_ would result in isotopic compositions consistent with the strongly depleted ^13^C values observed. At a minimum, however, new observations would be needed to confirm a biological origin for Mars CH_4_ plumes before this explanation can be accepted.

Alternatively, photochemical reduction of CO_2_ to formaldehyde (CH_2_O) via CO as an intermediate (e.g., ref. 64) might be responsible for producing a ^13^C-depleted organic material because the photochemical partitioning of CO_2_ and CO can lead to ^13^C depletion in the CO relative to CO_2_ (65–67). However, most past studies have considered Martian CO to be isotopically similar to CO_2_, with any ^13^C depletion relative to CO_2_ to be on the order of about 30‰ (68). Recently, however, photodissociation of CO has been shown to yield hundreds of per mil ^13^C enrichments in the resultant CO_2_ at 70 K using vacuum ultraviolet (VUV) radiation around 100 nm from a synchrotron source (69), while photodissociation of CO_2_ has been shown to yield tens of per mil ^13^C enrichments in the resultant CO (70, 71). While these experiments use different ultraviolet (UV) wavelengths and different temperatures, together they illustrate how photochemical partitioning in the Martian atmosphere might result in anomalously ^13^C-depleted CO that could influence geochemistry observed at the Martian surface (72). In fact, CO photolysis has been calculated to have large carbon isotopic fractionation (with a fractionation factor of 0.6) at the top of the Martian atmosphere (73). VUV radiation would interact with the upper Martian atmosphere, and the presence of CO in the Martian atmosphere is maintained by CO_2_ photolysis. If Martian photochemistry resulted in ^13^C-depleted CO throughout the atmosphere in the past, photoproduction of formaldehyde and other organics from CO could have accumulated on the exposed surface at Gale crater, resulting in the isotope results reported here (67, 74). Obstacles to the deposition of organic material from the photolysis of CO_2_ have been the low yield and photochemical instability of the formaldehyde produced (75). Photochemically produced formaldehyde on Mars might react quickly with SO_2_ to yield hydroxymethanesulfonate, protecting it from photolysis back to CO (76), and it is plausible that elevated levels of atmospheric SO_2_ would directly shield formaldehyde because of the spectral overlap of SO_2_ and CH_2_O photoabsorption cross sections combined with the greater strength of SO_2_ absorption (77, 78). After major eruptions, when there were higher levels of CO, SO_2_, and H_2_, the deposition of organics should be maximized, including perhaps formaldehyde, hydroxymethanesulfonate, carbonyl sulfide (79), and thioformaldehyde (80). Because of a relatively long photochemical equilibrium lifetime of SO_2_ of ∼1 Mars year (75, 81) and photodissociative shielding of CH_2_O afforded by SO_2_, the likelihood of such reactions increases. Incorporation of ^13^C-depleted organics into frost or glacial ice at the Martian surface could also concentrate these photochemical products and lead to their preservation in the local soil, protected from further UV reactions. However, the VUV wavelength radiation shown to produce large fractionation is too short for most of the Martian atmosphere and has yet to be explored at various temperatures, limiting any conclusions that can be drawn here. Also, direct observation of CO in the Martian atmosphere by spectroscopy from Earth has not revealed a large ^13^C depletion in CO, with the results appearing to be within 250‰ of the telluric value (82) and probably slightly ^13^C enriched relative to Earth (66, 68). Without further information with which to deconvolve the details of where and how carbon was fractionated, we can conclude the photochemical production of organic material from ^13^C-depleted CO is a possible scenario on Mars for deposition of highly depleted organic material on to an exposed surface (74) that should not be rejected without further investigation of Martian CO and the photochemical processes that influence its carbon isotopic composition.

## Conclusions

**Fig. 5.**
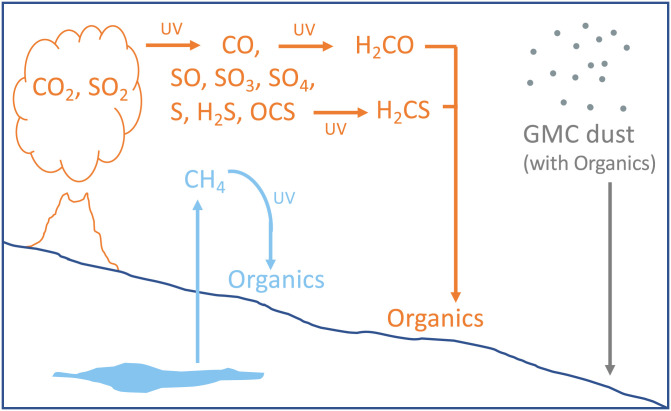
Three possible scenarios for the origin of the depleted carbon isotopes observed by the SAM TLS. Shown in blue, biologically produced methane from the Martian interior could result in deposition of ^13^C-depleted organic material after photolysis. While deposition of the depleted organics could also be due to methanotrophy, the paleosurface has not yet provided any evidence for the existence of methanotrophic mats. Shown in orange, photochemical reactions (UV) can result in various atmospheric products, some of which would be deposited as relatively labile organic material. It is unknown whether the photochemical reduction of CO_2_ to CO has a large isotopic fractionation under Martian conditions. In gray, ^13^C-depleted organic material would enter the Martian atmosphere if our solar system traversed a GMC.

There are multiple plausible explanations for anomalously ^13^C-depleted methane observed by the TLS portion of the SAM instrument suite during EGA of samples from multiple sedimentary horizons at Gale crater, Mars. With present knowledge, there are three options that are consistent with the isotopic values and geology observed. These consistent explanations include the photolysis of biological methane, photoreduction of CO_2_, and deposition of comic dust during passage through a GMC (Fig. 5). Although photochemical haze might have contributed to the Earth’s sedimentary record in the Archean, all three of these scenarios are unconventional, unlike processes common on Earth. With present knowledge, it will be difficult to determine which of the three scenarios most accurately depicts the events that unfolded on Mars billions of years ago. The *Curiosity* rover will again transect the Greenheugh pediment in the coming year, providing an opportunity to again sample this surface and document the chemical attributes of this landform and any organic carbon associated with it. Similarly, the association of the observed anomalous isotopic values with such an ancient erosion surface provides guidance for NASA’s *Perseverance* rover team for targeting samples that could provide additional insight into the Martian processes responsible for this isotopic fractionation.

## Methods

### Samples.

The first drilled MSL samples were collected from the Sheepbed member mudstones in Yellowknife Bay. The Yellowknife Bay region in Gale includes portions of high thermal inertia rocks toward the distal end of the alluvial fan from Peace Vallis. The first and second samples of Sheepbed mudstones—John Klein and CB, respectively—were taken to investigate rocks deposited in this fluvial-lacustrine context. Analyses of these samples indicated the presence of an ancient habitable environment on Mars (2).

After analysis of the Sheepbed mudstones, the rover continued its traverse through Aeolis Palus and then began its ascent of Mt. Sharp. Starting in September 2014, the rover transitioned from Bradbury group rocks to Mt. Sharp group rocks, which consist of hundreds of meters of sedimentary stratigraphy subdivided into the Murray formation and the Carolyn Shoemaker formation. The Murray formation comprises the basal unit of Mt. Sharp and is divided among several distinct lithologic members dominated by mudstones. The Carolyn Shoemaker formation conformably overlies the Murray formation and has prevalent sandstone lithology. To date, more than 20 drilled samples of Mt. Sharp group rocks have been collected and analyzed from the various members. Included among these samples are analyses of regions that show unique properties from orbit such as the VRR, the Glen Torridon region, and the lower sulfate-bearing unit. VRR is a prominent topographic high on lower Mount Sharp that had orbital evidence for elevated hematite and was determined to have formed from several diagenetic events (14). South of the ridge is the Glen Torridon region, which was notable for its elevated orbital spectral signature of phyllosilicates. The most recent samples (as of July 2021) have been documenting the transition from clays to hydrated sulfates.

In a few locations along the rover’s traverse, samples have been taken from the Siccar Point group, which is comprised of the Stimson formation sandstones. The Stimson formation unconformably overlies the Mt. Sharp group and represents the remnants of an ancient, lithified dune field. The first four samples of Stimson formation rocks were taken to study both the parent rock and areas of localized alteration. The fifth sample of Stimson formation was taken later in the mission, during the Glen Torridon campaign, when the rover ascended the Greenheugh pediment.

In addition to the drilled samples, MSL has analyzed scooped samples of sand. The fine sand-sized GB and Ogunquit Beach samples were taken from the active Bagnold Dune field and represent local modern aeolian processes.

### MSL EGA and MSL Carbon Isotopic Analysis.

The SAM consists of a suite of instruments including two pyrolysis ovens, a QMS, six gas chromatography columns, and a TLS (3). Together, SAM can measure the volatile composition of solid samples on Mars through thermal decomposition experiments. EGA–mass spectrometry (EGA-MS) heats samples to ∼850 °C and sends evolved volatiles directly to the QMS for identification with a constant flow of He. In this paper, QMS was used for the quantification of sulfur gases, as well as BSW (see *SI Appendix,* Supplementary Text for additional details). As explained further in the supplement, quadratic discriminant analyses of the QMS gas results provided posterior probabilities for whether each drill sample contained reduced sulfur (6). Gases during EGA can be sampled by the TLS within a specified temperature range. The TLS uses several specific infrared wavelengths to quantify H_2_O, CO_2_, and CH_4_ as well as the isotopes of these gases when present in great enough abundance.

For SAM EGA runs, the solid sample is contained in an oven whose temperature is increased at 35 °C/min to ∼850 °C while helium (0.8 sccm, 25 mbar) is flowed through the pyrolysis oven. After evacuation of the TLS sample (Herriott) cell to record empty cell values, the TLS cell is opened to receive a “temperature cut” ingest (e.g., 200 to 350 °C) during EGA that introduces He and evolved volatiles into the cell, which is then sealed off for analysis. Typically, empty-cell methane abundances are extremely low (parts per billion by volume) compared to the full-cell EGA values (tens of parts per million by volume [ppmv]) so that empty-cell corrections are not needed. Full-cell pressures (mainly helium) are typically 5 to 15 mbar.

As further discussed in the supplement, three strong ^12^CH_4_ lines and four strong ^13^CH_4_ CH_4_ lines are identified in the recorded TLS spectra, although only two of the ^13^CH_4_ lines are chosen for analysis to minimize water interferences (*SI Appendix*, Fig. S1). With the cell closed, the laser scans over the methane lines every second, and on-board, TLS captures average spectra over sequential 2.7-min periods that are downloaded. Analysis of each of these 2.7-min spectra is done on a line-by-line basis through comparison with HITRAN 2016 calculations (that employ terrestrial δ^13^C values, so that volume mixing ratios in ppmv are retrieved for each of the three ^12^CH_4_ and two ^13^CH_4_ lines. In some cases of very high mixing ratios, the strongest ^12^CH_4_ is very deep and not used. Then, the average values of the retrieved volume mixing ratios for ^12^CH_4_ and for ^13^CH_4_ are calculated from each of the 2.7-min spectra. During the run, 26 full-cell spectra are recorded, so that the 26 values retrieved of average abundance values can be statistically analyzed to find the mean value with SEs of either 67% CI or 95% CI. Ratioing the ^13^CH_4_ mean abundance with that determined for ^12^CH_4_ provides the δ^13^C values reported here normalized to the terrestrial PDB reference expectation such that

δ13C=((C13C12) Sample(C13C12) PBD std.−1) × 1,000 ‰,

where PDB std. is Pee Dee Belemnite standard, which is equal to 0.0112372.

### Supporting Laboratory Analyses.

As discussed in the *SI Appendix*,* Supplementary Text*, laboratory analyses were conducted (*SI Appendix*, Figs. S3 and S4, and Table S2) in support of this paper, including an NMR-based investigation (83) of the intramolecular isotopes of methyl-trifluoroacetamide, part of the MTBSTFA molecule. Laboratory studies were also used to study carbon isotopic fractionation during pyrolysis to CH_4_ under conditions similar to that of the SAM instrument. Various carbon-containing materials were placed in silver boats and dropped into an oven at 400 °C under flowing helium. The oven temperature then increased to >850 °C, and evolved gases, including CH_4_, were trapped out of the He flow between 455 °C and 755 °C to simulate a high-temperature TLS cut.

## Supplementary Material

Supplementary File

## Data Availability

All MSL SAM data are available at the Geosciences Node of NASA’s Planetary Data System (https://pds-geosciences.wustl.edu/missions/msl/sam.htm). All study data are included in the article and/or *SI Appendix*.
