# Supplementary material for: Depleted carbon isotope compositions observed at Gale crater, Mars
Source: Proc Natl Acad Sci U S A. 2022 Jan 18;119(4):e2115651119. doi: 10.1073/pnas.2115651119 (PMC8795525; doi:10.1073/pnas.2115651119)
Supplement: Supplementary File [file pnas.2115651119.sapp.pdf]

PNAS
www.pnas.org

Supplementary Information for
Depleted carbon isotope compositions observed at Gale crater, Mars.

Christopher H. House, Gregory M. Wong, Christopher R. Webster, Gregory J. Flesch, Heather B. Franz, Jennifer C. Stern, Alex Pavlov, Sushil K. Atreya, Jennifer L. Eigenbrode, Alexis Gilbert, Amy E. Hofmann, Maëva Millan, Andrew Steele, Daniel P. Glavin, Charles A. Malespin, Paul R. Mahaffy

Christopher H. House
Email: chrishouse@psu.edu

This PDF file includes:

Supplementary text
Figures S1 to S9
Tables S1 to S4
SI References

Additional detail on methods and additional SAM results at Gale Crater, Mars

Spectrum at 3.27 µm of evolved CH_4 from Yellowknife Bay, Gale Crater, Mars. For TLS analyses during EGA, CH_4 concentrations are typically about 1,000 times greater than the CH_4 signals typically observed in atmospheric measurements by TLS. The greater CH_4 abundances allow for isotopic measurements from $^{13}CH_4$ and $^{12}CH_4$. Fig. S1 shows, for example, the Cumberland 3 (CB3) TLS spectra from the 3.27 µm laser. The spectrum is exceptionally clean (e.g., with no background interfering lines) and provides multiple $^{12}CH_4$ and $^{13}CH_4$ lines with which to calculate $\delta^{13}C$ values. In practice, two of our $^{13}CH_4$ are used because they are well isolated, showing no spectral distortion in the second harmonic spectra, and they have no underlying interferences. One of the discarded lines shows spectral distortion at one lobe, and the second discarded line has a small water line underneath that becomes problematic at high water abundance. The 3 parent lines are always very well behaved except in very high CH_4 abundance when the g-line is very deep. Typically, therefore, we have at least four permutations of ratios of spectral lines (using the e, f, a, and d lines) that agree within a few permil of each other.

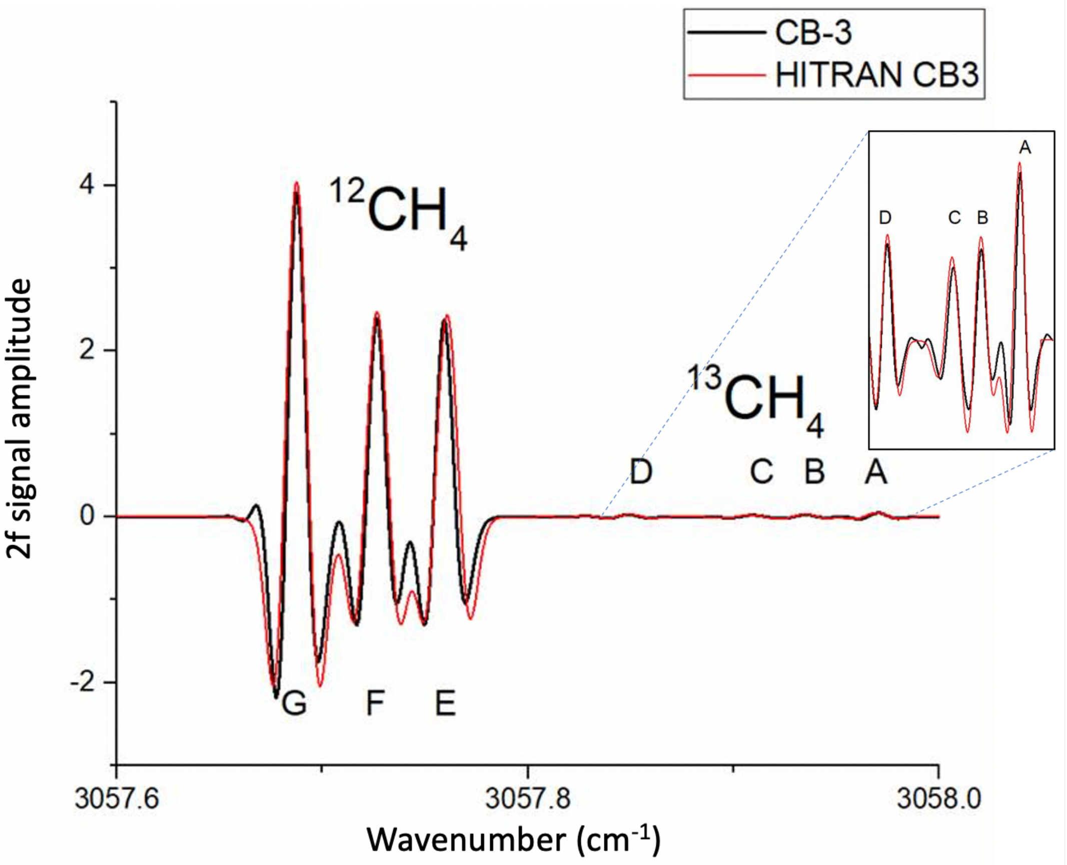

Figure S1. TLS second harmonic (modulated) spectrum generated from the on-board laser drive and signal chain electronics showing lines from CH_4 during the EGA analysis of CB3 (Black), compared to a HITRAN spectrum at the same scale (Red). CB3 is ~12 ppmv CH_4 in He, about 1,000 times the CH_4 signals typical of MSL atmospheric runs.

For retrieval of $^{12}CH_4$ and $^{13}CH_4$ abundances on a line-by-line basis prior to taking appropriate ratios, comparison is made with predictions from the HITRAN 2012 line parameter list that reports line strengths, ground-state energies, line assignments, self- and foreign-broadening coefficients. Because the original HITRAN line list reports foreign gas broadening by only air, we conducted meticulous laboratory measurements (1) of foreign broadening of the same a through g lines used by TLS that included the broadening gases air, CO_2, and helium, the latter being the buffer gas for the EGA measurements reported here. Measured He-broadening coefficients in cm-1/atm were typically ~70% of those for air-broadening of the TLS lines, and differences from the original HITRAN 2012 air-broadening coefficients were up to ~25%. These improved broadening coefficients were incorporated into our HITRAN calculations.

At the very low pressures (<15 mbar He) of the SAM EGA runs, line-broadening by He is very small (<3% of the line-width), and as the line is broadened, the intensity at the peak is distributed into the width and tails, so that the integrated line absorption ("area") remains constant. For TLS data analysis, for each line we integrate across the complete line and do the same across the improved HITRAN-generated lines for the comparison that produces final mixing ratios. Thus, our data processing method highly mitigates against errors in line broadening.

While all the $^{12}CH_4$ and $^{13}CH_4$ lines will have associated small systematic errors (e.g. pressure, pathlength, temperature) that we estimate could produce ~10 permil error in a single line result, these systematic errors are reduced in the isotope ratio determination because we scan over all the a through g lines simultaneously (once per second). The S.E. precision reported in this manuscript represents only the scatter in the point-by-point $\delta^{13}C$ retrievals at the 67% confidence interval, which is appropriated for comparison of delta values across many different samples studied by the same instrument in the same configuration and identical operational sequence script. For absolute values of our reported delta values, an uncertainty of twice the S.E. is recommended to represent the 95% confidence interval.

^{13}C-depleted evolved CO_2 observed in spectra from Yellowknife Bay, Gale Crater, Mars. Early in the mission, at Yellowknife Bay, the TLS analyses of Cumberland (CB) using the 2.78 μm laser produced CO_2 $\delta^{13}C$ values that are depleted to levels (Table S1) not observed in later samples of the mission. A couple of these anomalously ^{13}C-depleted CO_2 values (CB1 and CB6) are consistent with the TLS CH_4 isotopic values for the same sample run from the 3.27 μm laser. Enigmatically, the TLS CO_2 $\delta^{13}C$ values and the $\delta^{13}C$ values calculated from the EGA QMS, though, are not consistent with each other in a couple of cases (2). This enigma, and the correspondence between TLS CH_4 isotopic values and TLS CO_2 isotopic values for CB1 and CB6, makes it attractive to consider whether CH_4 could have oxidized to CO_2 in the TLS chamber. However, there is about 100 times more CO_2 than CH_4 rendering this explanation challenging. Further research is needed to elucidate the meaning and origin of the CB TLS CO_2 isotopic values, including a detailed analysis of the CB combustion experiments (3). Because the highly depleted CH_4 $\delta^{13}C$ values from the from 3.27 μm laser return several times during the mission, we have focused on those results for this study.

Table S1. MSL CO_2 Isotopic Values from EGA analyses of CB.

Label	Sol	Temp. Cut for TLS (˚C)	CO_2 (nmoles)	±S.E.	TLS CO_2 $\delta^{13}C$ (‰)	± 1 S.E.	Notes
CB1	281	220 to 319	645.7	3.9	-73	5.4	Consistent with evolved CH_4 isotopes. *
CB2	286	99 to 349	1012.5	5.8	-256	5.6	Substantially more ^{13}C-depleted than evolved CH_4 isotopes. *
CB5	368	450 to 786	402.5	2.3	-18	11.7	Cup pre-treated to 200 ˚C; Consistent with Mars igenous carbon.
CB6	382	450 to 786	436.1	2.5	-76	9	Cup and sample pre-treated to 250 ˚C; Consistent with evolved CH_4 isotopes.
CB7	415	156 to 443	617.1	2.4	-28	7	Cup and sample pre-treated to 250 ˚C; Consistent with Mars igenous carbon; No CH_4 isotopes for this run.

Note: * SAM QMS value (2) for this sample is not as ^{13}C-depleted.

N-tert-butyldimethylsilyl-N-methyltrifluoroacetamide (MTBSTFA) Instrument Background. Due to an early leak of the derivatization agent, N-tert-butyldimethylsilyl-N-methyltrifluoroacetamide (MTBSTFA), in the Sample Manipulation System (SMS) and residual MTBSTFA from subsequent derivatization experiments, it is available as a potential source of non-indigenous carbon (4). Therefore, MTBSTFA during EGA was tracked to assess its influence on methane abundance and its carbon isotope values. As one measure of the amount of MTBSTFA background found in each EGA experiment, we have quantified and reported here the total observed 1,3-bis(1,1-dimethylethyl)-1,1,3,3-tetramethyldisiloxane (or bi-silylated water, BSW) which is formed from the reaction of MTBSTFA with water in the SAM oven during the experiment. BSW was monitored during EGA using the QMS portion of SAM. BSW abundance is related to several factors; principally, it is a function of MTBSTFA abundance, sample surface area, and amount of time the sample was exposed to MTBSTFA. BSW is observed in all EGA runs and typically evolves below 500 ^{o}C (5). While BSW may not directly contribute carbon for methane isotopes, particularly at high temperature, it remains a convenient and useful tracker for overall MTBSTFA contamination in a given sample.

Sulfur analyses. Additionally, sulfur redox chemistry was considered based on quadratic discriminant analysis (QDA) of EGA sulfur volatiles and sulfur isotopes as described previously (6–8). Sulfur QDA results that had not been previously reported were calculated using the same methods described in Wong *et al.* (6). Briefly, quadratic discriminant analysis is a classification method that compares data from unknown samples (select volatiles from EGA of Martian samples) to data from known samples used to create a training dataset (the same volatiles from SAM-like EGA laboratory analogues). In this case, laboratory samples were of known composition and either contained sulfide or sulfate. Evolved SO_2, COS, CS_2, CO_2, and BSW were used as variables in QDA. Based on the training dataset, the prior probability of a sulfide was calculated to be 0.61 (34 samples containing sulfide out of 56 total training samples) (6, 9). The posterior probability (PP) was calculated as a function of the prior probability and the class-conditional distributions of evolved volatiles. Mars samples were considered to contain a sulfide if the posterior

probability was greater than 50% (6). QDA analyses were performed in Python 2.7 using the Scikit-learn package (10).

Sulfur isotopes were calculated according to the methods described in Franz *et al.* (7). Briefly, the reported $\delta^{34}S$ values were calculated from evolved SO_2 during EGA. Ratios of $^{34}SO_2$ to $^{32}SO_2$ were calculated for the major Fe-sulfate/sulfide peak (~550 °C). After correcting for isobaric interferences, the $\delta^{34}S$ values were reported compared to the V-CDT standard.

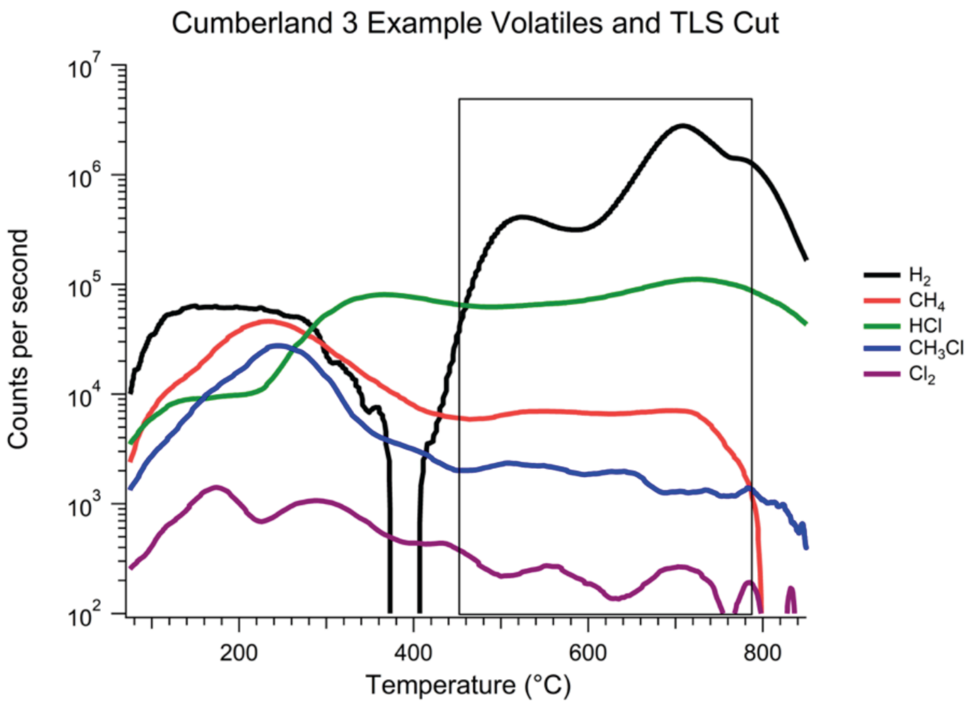

Figure S2. EGA plot showing counts per second vs. temperature in degrees Celsius for five example volatiles. The box marks off the temperature range 450˚C – 786˚C used for the TLS cut.

Select volatiles released during EGA at Yellowknife Bay, Gale Crater, Mars. For illustration purposes, a few select volatiles from EGA are plotted in Fig. S2 along with the temperature cut during which gases were sent to the TLS (in addition to the QMS) for analysis. Later in this supplement, reactions between CH_4 and chlorinated compounds will be considered as a possible mechanism for producing ^{13}C-depleted methane in the pyrolysis oven (Figs. S5-S8). Hence, the plot focuses on evolved CH_4, H_2, HCl, Cl_2, and CH_3Cl. Figure S2 shows the relative amounts of each gas with $[H_2] > [HCl] > [CH_4] > [CH_3Cl] > [Cl_2]$ in the temperature cut. During EGA of CB3, CH_4 evolved as a substantial peak with its maximum centered at about 240°C followed by a relatively flat continual CH_4 release until dropping at a temperature above 720°C (when the largest H_2 release also approximately begins to decline). Broadly, CH_3Cl has a similar release profile to the CH_4 release in this case.

Details about supporting laboratory analyses

Intramolecular Isotopes of methyl-trifluoroacetamide from the hydrolysis of MTBSTFA. The bulk $\delta^{13}C$ value for the MTBSTFA that constitutes the SAM background is -35‰. Because they constitute the majority of the carbon in MTBSTFA, the methyl groups are likely to be isotopically similar to the bulk carbon isotopic value of the molecule and unlikely to release highly ^{13}C-depleted methane upon simple methyl cleavage. However, the carbonyl carbon of MTBSTFA would need to undergo a significant reduction to produce CH_4 in the pyrolysis oven. As will be discussed later, carbonyl carbon reduction to CH_4 can result in tens of permil carbon isotopic fractionation. Even if there were a set of conditions where pyrolysis release was predominately from the reduction of the MTBSTFA carbonyl carbon, which seems unlikely, tens of permil isotopic fractionation is not large enough to explain the observations reported here for evolved CH_4 at several locations in Gale crater, Mars. In principle, with its distinct oxidation state and chemistry, the carbonyl carbon of MTBSTFA could have a unique site-specific carbon isotopic value far from the bulk of the molecule. If so, it is conceivable that under certain oven conditions, this specific carbon could contribute disproportionally more carbon to the evolved CH_4 accounting for the occasional observation of anomalous CH_4. For this to work as an explanation for the results observed, the carbonyl carbon of MTBSTFA would need to be significantly more ^{13}C-depleted than other carbons in the molecule.

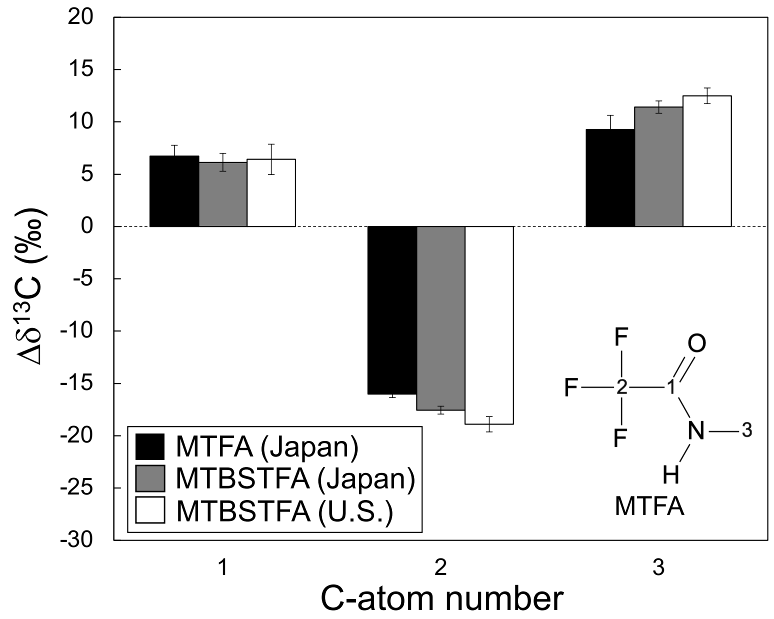

Figure S3. Three intramolecular isotopic NMR analyses of methyl-trifluoroacetamide (MTFA). Purchased MTFA shown in black, MTFA derived from MTBSTFA purchased in Japan is shown in grey, and MTFA derived from MTBSTFA purchased in the United States is shown in white. All three samples are in agreement that the trifluorinated carbon (labeled as 2) is most ^{13}C-depleted.

We tested this possibility through an NMR study of methyl-trifluoroacetamide (MTFA) purchased through Sigma Aldrich, as well as MTFA derived from MTBSTFA reagents purchased through Sigma Aldrich, United States, and Sigma Aldrich, Japan. Briefly,

MTBSTFA was reacted in an excess of methanol producing MTFA and tert-butyldimethylsilyl methoxide (TBDMS-OMe) in equimolar amounts. The excess methanol and TBDMS-OMe formed were evaporated under vacuum, leading to MTFA in good yield (95%) and purity (>99.5%; assessed by ^1H NMR). Carbon isotopic analysis was conducted at Tokyo Institute of Technology in DMSO-d_6 following Gilbert et al. (11). Carbon isotopic analysis via NMR yields relative isotopic differences between sites within a molecule (shown in Fig. S3 as a $\Delta^{13}C$). The results (Fig. S3) show the fluorinated carbon, labeled 2, is ^{13}C-depleted by up to 25‰ relative to the other two carbons, including the carbonyl carbon. This level of depletion, while large, is unlikely to be the source of the anomalously lower evolved CH_4 $\delta^{13}C$ values reported here because this carbon is the least likely to evolve ample CH_4 during pyrolysis due to its three fluorine bonds. The types of carbon (methyl-carbon and carbonyl C) most likely to contribute CH_4 from the MTBSTFA background are actually the most ^{13}C-enriched. The methyl carbon, labeled 3, is the most ^{13}C-enriched component of MTFA, but is still within 10‰ of the carbonyl carbon. Given that the methyl carbon is probably close in carbon isotopic composition of the bulk MTBSTFA, this minor difference in ^{13}C composition observed between the carbonyl carbon and the methyl carbon may contribute to some variations observed for evolved methane; however, the intramolecular heterogeneity is not large enough to explain the anomalous ^{13}C-depletions reported here.

Laboratory results for carbon isotopic fractionation during pyrosis of various materials. Laboratory studies were conducted to estimate the range of carbon isotopic fractionation expected from pyrolysis. These experiments were aimed at establishing the degree to which the $\delta^{13}C$ CH_4 values could become ^{13}C-depleted due solely to carbon isotopic fractionation during the pyrolysis process. Various carbon-containing materials were placed in silver boats and dropped into the 400 °C oven of a repurposed elemental analyzer under flowing helium (120 mL/minute; 804-838 Torr). The oven temperature then rose to >880°C at a rate of about 12°/minute), and the flow was diverted through a molecular sieve-containing stainless-steel trap in liquid N_2 when the oven temperature was between 455 °C and 755 °C to simulate a high temperature TLS cut (similar to CB3). In some cases, the sample trap was then removed from the system and warmed to room temperature. In other cases, the trap was evacuated on the gas line while cold, then expanded into the gas space and refrozen onto the trap. In the latter cases, helium was added to the trap before it was removed from the gas line. In all cases except with methylphosphonic acid (MP1), the trap was filled with helium when the trap was cold so that there would be slight overpressure after warming. In the case of MP1, when the trapped gases were expanded into a pressure gauge on the stainless-steel trap by warming, it showed a significant positive pressure, and so no helium was added after the gas mixture was re-trapped. Several days after the sample traps were warmed to room temperature, they were syringe sampled for CH_4 concentration (via gas chromatography) and CH_4 $\delta^{13}C$ composition (via gas chromatography-isotope ratio mass spectrometry with an inline oxidation oven). Additionally, the starting materials were analyzed for their bulk carbon isotopic composition by an Elemental Analyzer Isotope Ratio Mass Spectrometer (EA-IRMS). All of these analyses were performed by the Penn State Laboratory for Isotopes and Metals in the Environment (LIME).

The results (Table S2) include approximations for the carbon isotopic fractionation

factor (α) during the pyrolysis of different carbon-containing materials and the isolation of evolved gases via a flow diversion during a specific temperature cut (445 °C - 755 °C). It is perhaps important to consider that this dataset includes different fractionation processes than those on SAM. Specifically, in this work, there are materials that have quite labile methyl groups, as well as molecules with only highly recalcitrant carbon. There are also a range of carbon oxidation states represented in the dataset.

Table S2. Supporting pyrolysis laboratory experiments data.

Label	Material	C component mass (mg)	M.W.	#C per molecule	C mass (mg)	Bulk $\delta^{13}C$	$\delta^{13}C$ methane	Fractionation factor	1000* ln α
062716_TS1	tetra (trimethlsilyl)silane	25.6	320.8	12	11.51	-36.3	-40.7	0.9954	-4.6
062816_blk2	Silver boat blank	0.0	N/A	N/A	N/A	N/A	-41.6		
062816_MPI	Methylphosphonic acid	25.2	96.0	1	3.15	-101.6	-102.0	0.9996	-0.4
121516 CO3 FeNi1	Sodium Bicarbonate + FeNi	10.6	84.0	1	1.52	-6.4	-34.4	0.9719	-28.5
121516 CO3FeSS1	Sodium Bicarbonate + FeS_2	10.4	84.0	1	1.49	-6.4	-54.0	0.9521	-49.0
121616 CO3 FeNi2 X2	Sodium Bicarbonate + FeNi	20.7	84.0	1	2.95	-6.4	-38.1	0.9681	-32.4
121616 CO3FeSS2	Sodium Bicarbonate + FeS_2	27.6	84.0	1	3.94	-6.4	-47.9	0.9583	-42.6
121916 G3	Graphite	20.4	NA	NA	20.41	-26.6	-34.5	0.9919	-8.1
122016 Blank 7	Silver boat blank	N/A	N/A	N/A	N/A	N/A	-40.3		
010417_Ph4_3x	Phenanthrene	76.5	178.2	14	72.13	-24.5	-47.1	0.9768	-23.4
010517_TS2	tetra (trimethlsilyl)silane	24.8	320.8	12	11.13	-36.25	-38.6	0.9976	-2.4
021918 OXLT_FeSS2	Sodium Oxalate + FeS_2	10.7	134.0	2	1.92	-24.9	-49.25	0.9750	-25.3
0222118 OXL_FeNi	Sodium Oxalate + FeNi	10.8	134.0	2	1.93	-24.9	-50.7	0.9735	-26.8
022118-BLK	Silver boat blank	N/A	N/A	N/A	N/A	N/A	-38.65		
0222118 OAM_FeNi	Oxamide + FeNi	2.1	88.1	2	0.58	-19.7	-44.2	0.9750	-25.3
022618 OAMFeSS2	Oxamide + FeS_2	2.3	88.1	2	0.63	-19.7	-43.95	0.9753	-25.0
041018_blank (with leak)	Silver boat blank & Lab Air	N/A	N/A	N/A	N/A	N/A	-50.2		
050718_G_FeSS_SiO_2x_1	Graphite + FeS_2+ silicic	20.0	N/A	N/A	20	-26.6	-47	0.9790	-21.2
050718_Dia_FeSS_SiO_2x_1	Diamond + FeS_2 + silicic	20.0	N/A	N/A	20	-30.2	-44.1	0.9857	-14.4
050818_blank	Silver boat blank	N/A	N/A	N/A	N/A	N/A	ND		
051818_formic_H2O_2x	Formic acid + water + SiO_2	20.0	46.0	1	20.00	-32.6	-47.7	0.9844	-15.7

Helium added to Sample trap (Torr)	He in sample trap (atm)	Sample trap #	Sample trap volume (mL)	Approx. total gas (mL)	CH_4 ppm (from GC)	mL CH_4	L CH_4	moles CH_4	Gas production	Fractional C yield (ppm)
796	1.05	1	15	15.7	84.0	0.001320	1.32E-06	5.89E-08	Substantial	6.1E+01
786	1.03	1	15	15.5	0.2	0.000004	3.57E-09	1.59E-10	N/A	N/A
N/A	~2	1	15	~30	154.0	0.004620	4.62E-06	2.06E-07	Major	~800
773	1.02	4	13	13.2	1.7	0.000022	2.25E-08	1.00E-09	Trace	7.9E+00
775	1.02	2	14	14.3	3.6	0.000051	5.14E-08	2.29E-09	Trace	1.9E+01
773	1.02	6	11	11.2	7.8	0.000087	8.73E-08	3.90E-09	Trace	1.6E+01
775	1.02	5	12	12.2	3.2	0.000039	3.92E-08	1.75E-09	Trace	5.3E+00
780	1.03	3	14	14.4	0.3	0.000004	4.31E-09	1.92E-10	Very Trace	1.1E-01
774	1.02	1	15	15.3	ND	ND	ND	ND	N/A	N/A
737	0.97	5	12	11.6	3.6	0.000042	4.19E-08	1.87E-09	Trace	3.1E-01
745	0.98	1	15	14.7	54.6	0.000803	8.03E-07	3.58E-08	Substantial	3.9E+01
746	0.98	2	14	13.7	1.7	0.000023	2.34E-08	1.04E-09	Trace	6.5E+00
748	0.98	5	12	11.8	1.1	0.000013	1.30E-08	5.80E-10	Trace	3.6E+00
748	0.98	3	14	13.8	0.3	0.000004	4.13E-09	1.84E-10	N/A	N/A
751	0.99	4	13	12.8	1.1	0.000014	1.41E-08	6.30E-10	Very Trace	1.3E+01
750	0.99	6	11	10.9	1.0	0.000011	1.09E-08	4.85E-10	Trace	9.3E+00
745	0.98	3	14	13.7	2.1	0.000029	2.88E-08	1.29E-09	N/A (Lab air)	N/A
741	0.98	5	12	11.7	2.4	0.000028	2.81E-08	1.25E-09	Trace	7.5E-01
740	0.97	1	15	14.6	2.4	0.000035	3.51E-08	1.56E-09	Trace	9.4E-01
738	0.97	3	14	13.6	1.0	0.000014	1.36E-08	6.07E-10	N/A	N/A
740	0.97	6	11	10.7	4.4	0.000047	4.71E-08	2.10E-09	Minor	1.3E+00

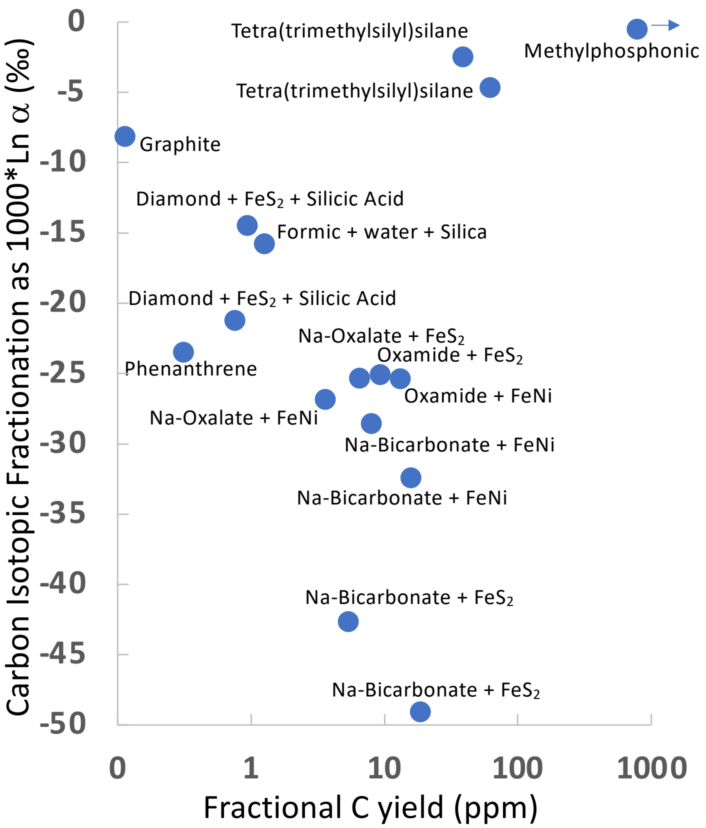

Figure S4. Carbon isotopic fractionation during pyrolysis of carbon-containing materials. The yield for methylphosphonic acid is more uncertain than other experiments due to the larger evolution of gases from that material. The fractional yield reported represents a minimum of the total CH_4 generated in each experiment because the results do not include untrapped CH_4 released below 455°C. This detail is most relevant for methylphosphonic, where significant gas production occurs at lower temperatures and where quantification was approximate. Similarly, the $\delta^{13}C$ values correspond only to the trapped gas (455 to 755°C).

Fig. S4 summarizes the carbon isotopic fractionation observed during these SAM-like pyrolysis experiments using different starting material mixtures. Overall, the results show a range of carbon isotope fractionation magnitudes (0.4 to 49‰) broadly consistent with thermogenic CH_4 production (12), considering that thermogenic CH_4 is derived from already ^{13}C-depleted organic material. As expected, materials with highly labile methyl groups (represented here by tetra(trimethylsilyl)silane and methylphosphonic acid) yielded ample evolved CH_4 with little ^{13}C depletion (<5‰). These results best approximate what likely happens when methyl groups from MTBSTFA form methane during SAM EGA. In contrast, a group of recalcitrant materials produced moderate carbon isotopic fractionations (8 to 21‰) to CH_4 with quite low CH_4 production (represented here by graphite and diamond). First with graphite, we found added silicic acid (hydrated silica) promoted CH_4 production potentially via hydrolytic processes during the pyrolysis. The production of CH_4 with a moderate level of ^{13}C-depletion due to pyrolysis could represent possible degrees of fractionation to be expected from the production of CH_4 from highly recalcitrant carbon indigenous to Mars, such as the igneous carbon previously reported from Martian

meteorites. We only found large carbon isotopic fractionation (28 to 49‰) during cases where inorganic carbon was reduced to CH_4, a result that appears supported by past laboratory hydrothermal experiments (discussed later). Additionally, there were several experiments that produced apparent fractionations around 25‰ (23 to 27‰). In all of these cases, the carbon in the original material had at least one carbon-carbon bond, but the materials represent disparate redox states for organic carbon with oxalate having relatively two oxidized carbonyl carbons and phenanthrene being more reduced. The similarity of these results from seemingly disparate starting organic molecules might suggest that carbon-carbon bond cleavage facilitates the required reduction to CH_4 of the resultant fragments. Alternatively, the similarity of these results might be coincidental as phenanthrene is quite volatile and could have mostly been mobilized prior to the collection of gases starting at 445 °C. If phenanthrene migrated to a location in the system with a considerably lower temperature, the collected CH_4, in that case, would represent CH_4 production under different conditions.

Overall, the results of our laboratory studies indicate that pyrolysis like that performed by the SAM instrument can produce up to about 50‰ carbon isotopic fractionation. The largest fractionations are expected when the starting material is inorganic carbon. The most extreme scenario would entail starting with Martian carbonate that has the igneous Martian $\delta^{13}C$ value of about -20‰. In that case, the maximum carbon isotopic fractionation of 50‰ could produce an evolved CH_4 $\delta^{13}C$ of about -70‰, a value that is still not as ^{13}C-depleted as several observations over the course of the MSL mission. However, given that Martian atmospheric carbon has a pronounced ^{13}C-enrichment (and likely has been ^{13}C-enriched for some time), highly ^{13}C-depleted evolved CH_4 being derived from Martian carbonates is unlikely. Other scenarios include the carbon isotopic fractionation of recalcitrant Martian igneous carbon or Martian organic material. In these cases, the resultant TLS CH_4 $\delta^{13}C$ values observed might be approximately 25‰ more ^{13}C-depleted than the original Martian carbon. In such scenarios, Yellowknife Bay, Vera Rubin ridge, and the Greenheugh pediment appear to still contain anomalously ^{13}C-depleted values, but the pretreated CB samples do not necessarily belong to this collection of results.

Modeling results for carbon isotopic fractionation during pyrolysis reactions

Formation of CH_3Cl and CH_4. Several reactions are possible to form methane or chloromethane during heating. Navarro-González et al. (13) determined with a chemical kinetics model that CH_4 and CH_3Cl are readily produced from a precursor organic carbon in Viking-like heating conditions. Here, we investigated a few example reactions in terms of their thermodynamic equilibrium constants as functions of temperature and their standard Gibbs free energies of reaction. We focused here on the potential for an equilibrium fractionation because a collection of kinetic fractionations forming halogenated organics from MTBSTFA-derived methane would be predicted to leave the unreacted methane ^{13}C-enriched (which is the opposite of what would be needed to explain the observed data). The reactions and their standard Gibbs free energies considered here are listed in Table S3.

Table S3. List of reactions and their standard Gibbs free energies explored in this work.

Reaction #	Reaction	ΔG° (kJ/mol)
1	$2CH_3 + Cl_2 \leftrightarrow 2CH_3Cl$	-411
2	$2CH_3 + H_2 \leftrightarrow 2CH_4$	-397
3	$2CH_3 + HCl \leftrightarrow CH_4 + CH_3Cl$	-308
4	$CH_4 + Cl_2 \leftrightarrow CH_3Cl + HCl$	-102
5	$CH_4 + HCl \leftrightarrow CH_3Cl + H_2$	89
6	$2CH_4 + Cl_2 \leftrightarrow 2\,CH_3Cl + H_2$	-13

Reactions 1-3 use CH_3 as the carbon reactant. CH_3 may be derived from the decomposition of larger organic compounds, methoxyl-bearing organics, or MTBSTFA, and it could form CH_4 and CH_3Cl (13–15). Reactions 1-3 support these hypotheses – production of methane and chloromethane is highly favored from CH_3 under standard conditions. Furthermore, the formation of CH_3Cl and CH_4 from these reactions is strongly favored at a wide range of temperatures, including those reached in the SAM oven (Fig. S5).

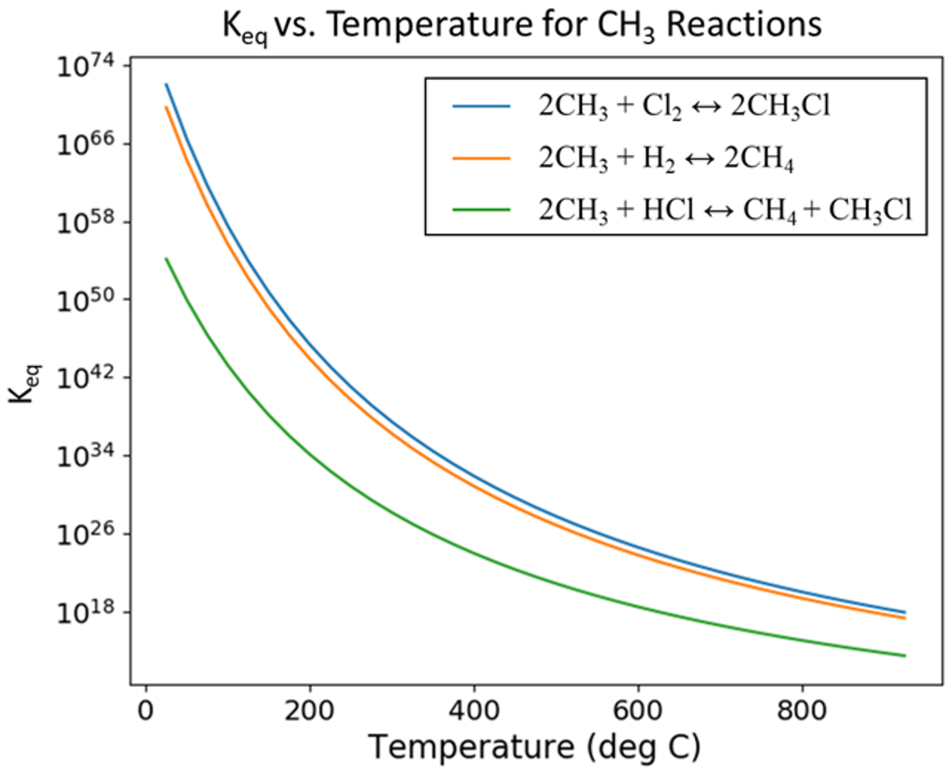

Figure S5. Equilibrium constants as functions of temperature for Reactions 1-3 in Table S3 (with CH_3 as the reactant). The equilibrium constants indicate that products (methane and chloromethane) are strongly favored over the entire SAM temperature range for these three reactions.

Reactions 4-6 in Table S3 investigate the relationships between methane and chloromethane more directly. Reaction 6 is the net reaction of Reactions 4 and 5. This net

reaction has a ΔG° of -13 kJ/mol, suggesting that the favorability of reactant vs. product formation has a relatively strong dependency on the presence of constituent volatiles and the temperature. CH_3Cl and H_2 are favored throughout the SAM oven temperature range, though only to a small degree (Fig. S6).

Together, these possible reactions set up a range of possibilities for methane and chloromethane formation during SAM analyses. Reactions 1-3 in Table S3 suggest that CH_3Cl and CH_4 would be readily formed by reactions involving CH_3 produced during decomposition of organic precursors. The formation of methane and chloromethane would be favorable in essentially all temperature conditions during pyrolysis. These gases could also react with HCl, Cl_2, and H_2, where the favorability of formation would be determined by temperature and relative volatiles available for reaction. Depending on the gases present, their flow, and time, methane and chloromethane may be able to equilibrate (Reaction 6).

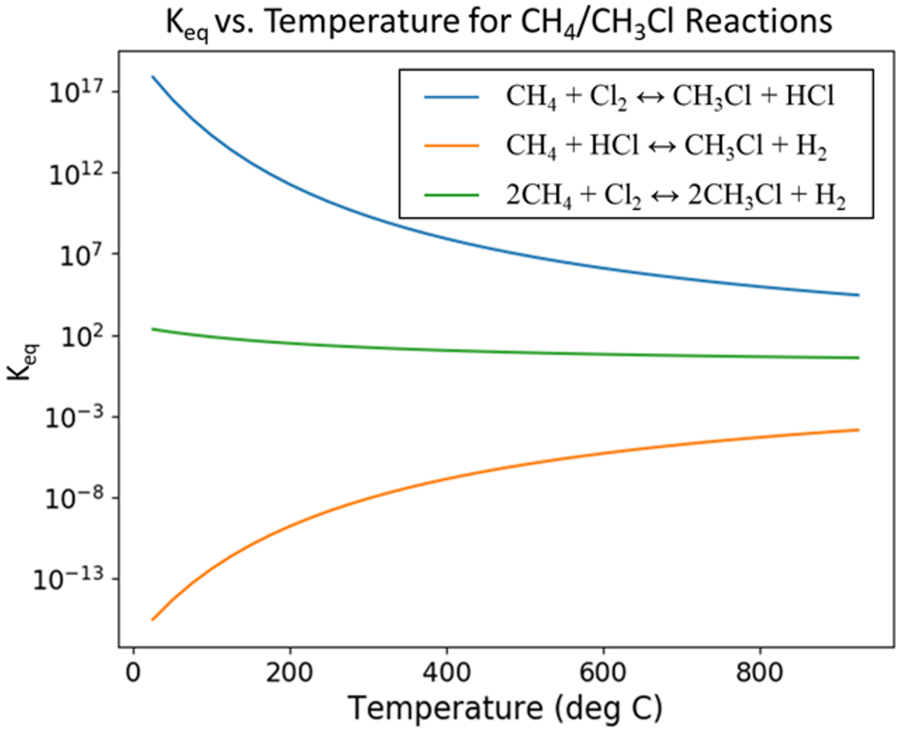

Figure S6. Plot of K_{eq} vs. temperature for Reactions 4-6 (methane to chloromethane conversion).

Kinetic fractionation. The results of isotope fractionation modeling for an irreversible reaction from precursor carbon to chloromethane are shown in Fig. S7. For possible fractionation factors, it was assumed that the precursor carbon would behave like methoxyl-functional group carbon as investigated by Keppler *et al*. (14). They investigated the isotopic effects of pyrolyzed meteorite material mixed with chlorine-bearing compounds and found that chloromethane had $\Delta^{13}C$ (defined as $\delta^{13}C_{precursor}$-$\delta^{13}C_{product}$) between -10 and -30‰ relative to native methoxy-bearing organics. Therefore, fractionation factors $\alpha_{P/R}$ of 0.99 and 0.97 were used, which can result in fractionation of the chloromethane by up to 10‰ and 30‰, respectively, lower than the precursor carbon

depending on the CH_3Cl yield. Chloromethanes typically comprise only a small fraction of the carbon volatiles observed during Martian pyrolysis experiments (4, 16). Small yields of CH_3Cl from a precursor carbon would result in large relative depletions in $\delta^{13}C$. Assuming a yield of 20% CH_3Cl from a precursor carbon, the $\Delta^{13}C$ of CH_3Cl would be ~ -9‰ for α=0.99 and ~ -27‰ for α=0.97.

An additional kinetic fractionation may occur during the formation of methane from chloromethane; however, such an effect is likely to be small. Taking the pyrolysis results for methylphosphonic acid (Table S2) and assuming similar behavior in chloromethane, methane can readily form in high abundance with a small fractionation of approximately -0.4‰ compared to the starting material. Taken together, if a precursor carbon were to form methane through this "chloromethane pathway," the resultant methane would likely be depleted by no more than 30‰.

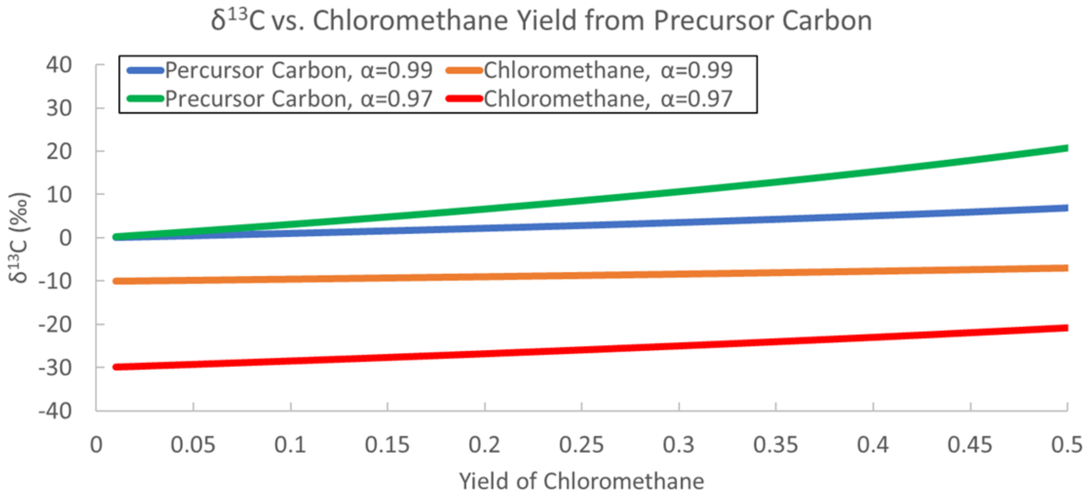

Figure S7. Irreversible Reaction Precursor C-->CH_3Cl with $\delta^{13}C_{VPDB}$=0‰ starting material, α=0.99 or 0.97. Red and orange lines indicate the resulting $\delta^{13}C$ in CH_3Cl for α=0.99 and 0.97, respectively.

Equilibrium fractionation between methane and chloromethane. Depending on the conditions of any given pyrolysis experiment, chloromethane and methane could equilibrate and result in important isotopic effects, which are generally modeled in Fig. S8. For this fractionation modeling, it was assumed that a reversible reaction between methane and chloromethane with a total $\delta^{13}C$ of 0‰ would occur in a closed system. An assumed fractionation factor $\alpha_{CM/Methane}$ of 1.018 was used, resulting in the relative depletion of methane. This equilibrium fractionation factor is based on calculations by Gropp, Iron, and Halevy (17) for the equilibration of CH_3OH and CH_4 at 50 ˚ C in enzyme-catalyzed biological systems. Methanol was chosen as an approximation for chloromethane in the absence of chloromethane fractionation data due to the similarity of -Cl and -OH functional groups. The redox similarity of the two molecules may make them fractionate similarly with methane. However, CH_3Cl has a longer C-Cl bond length (1.785 Å) compared to the C-O bond length (1.427 Å) in CH_3OH (18). The longer bond length in CH_3Cl may result in smaller fractionations due to the smaller relative difference between ^{13}C and ^{12}C. Indeed, laboratory investigations of abiotic reduction of chlorinated hydrocarbons have observed small fractionations on the order of 5‰ (19).

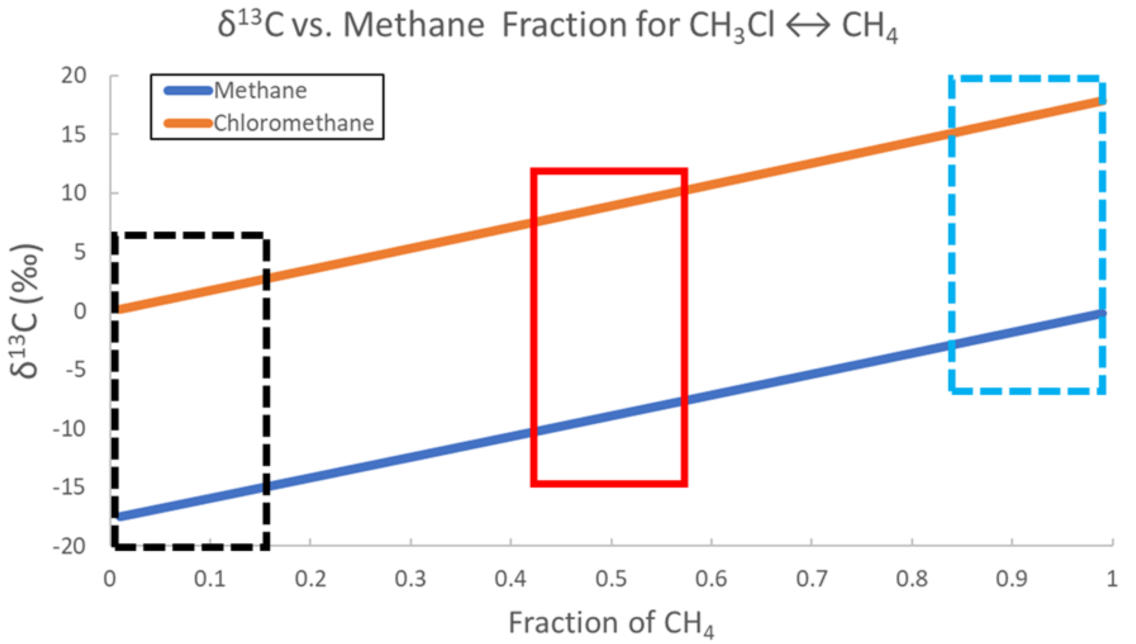

Figure S8 Carbon isotopic effects of a reversible reaction in a closed system for the equilibration of $CH_3Cl \leftrightarrow CH_4$ with an overall isotopic composition of $\delta^{13}C_{VPDB}$ = 0‰ (e.g., the composition of the original pyrolyzed carbon) with $\alpha_{CM/Methane}$ = 1.018. Boxes represent three different equilibration scenarios. The red box represents the instantaneous equilibration of CH_3Cl and CH_4 after their formation where they are present in equal abundances. Dashed boxes represent more extreme cases of equilibration with the black box (left) representing a more oxidizing environment and the blue box (right) representing a more reducing environment.

A few equilibration scenarios are explored here with the assumptions described above. In a case where CH_3Cl and CH_4 instantaneously equilibrate after their formation from a precursor carbon and are present in equal amounts, the resulting isotopic compositions would deviate by ~ +9‰ and ~ -9‰, respectively (Fig. S8, red box). In a case where methane and chloromethane were able to equilibrate according to the reaction $2CH_4 + Cl_2 \leftrightarrow 2CH_3Cl + H_2$ (Reaction 6 in the previous section), their relative abundances would be under redox control. In a more reducing environment, methane would be the favored carbon compound and would not have a large isotope deviation while CH_3Cl would be enriched by nearly 20‰ (Fig. S8, blue dashed box). Alternatively, in a more oxidizing environment, CH_3Cl would be favored and the small amounts of methane could be depleted by nearly 20‰ relative to the chloromethane (Fig. S8, black box). These scenarios may be largely sample-dependent, especially if redox controlled. However, the fractionation will be small in any system dominated by methane, which is the case for methane and chloromethane observed by TLS in which the fraction of methane vs. chloromethane is typically >0.7.

It is important to note that the scenarios described above only consider methane that forms through a pathway that includes chloromethane and do not include other sources of methane. A final TLS measurement could include methane from various sources including the sample, oven reactions, or cleavage from MTBSTFA ($\delta^{13}C$ = -35‰). These would mix

and affect the observed isotopic value. For example, methane readily forms from cleaved methyl groups from MTBSTFA (Fig. S4) with only small observed fractionation (~5‰ depletion). Such methane could constitute a large fraction of the observed MTBSTFA-derived methane and may have $\delta^{13}C$ = -40‰, which would mix with more depleted methane from the chloromethane pathway and reduce the magnitude of an observed depletion. Assuming a mixture of 50% methane from MTBSTFA methyl cleavage ($\delta^{13}C$ = -40‰) and 50% from a more extreme case of MTBSTFA-derived methane through the chloromethane pathway ($\delta^{13}C$ = -65‰), the observed $\delta^{13}C$ for methane would be –52.5‰. Such a value is much more enriched (~20-80‰) than the "highly-depleted" values discussed in the main text.

After using methanol as a basis for a conservative way of estimating fractionation, additionally, the equilibrium carbon isotope fractionation factor for the carbon exchange reaction between chloromethane (CH_3Cl) and methane (CH_4) was calculated directly from reduced partition function ratios via the Urey-Bigeleisen-Mayer approach (20, 21) using harmonic vibrational frequencies calculated from experimental data for the ^{12}C- and ^{13}C-bearing isotopologues of the two molecules (22, 23). The resulting alpha value ($^{13}\alpha_{CH3Cl-CH4}$) of 1.002 (calculated at 50°C) indicates that, if the two molecules are in isotopic equilibrium, methane will be depleted in ^{13}C by only ~2‰ relative to chloromethane.

Table S4. Endmember carbon isotope values on Earth and Mars. Ranges from Leshin *et al.* (24) and references therein except where otherwise noted.

**Carbon source**	**Approximate range of $\delta^{13}C$ (‰, V-PDB)**
Martian meteorite carbonate	+10 to +65
Martian refractory carbon	-28 to -5
Martian magmatic carbon	-30 to -20
Atmospheric CO_2 from SAM	+42 to +50
Bulk carbonaceous chondrites	-25 to 0
Terrestrial reduced carbon	-80 to -15
CO_2 from SAM EGA of Martian samples[a]	-25±20 to +56±11

[a]Values from Franz et al. (2).

Literature carbon isotopic fractionation results during reduction of CO_2. The largest carbon isotopic fractionations observed during our pyrolysis experiments were the results from reduction of inorganic carbon to CH_4. The maximum magnitude of carbon isotopic fractionation observed in our experiments (up to 50‰; Fig. S9A) are well in line with past research where either CO_2, CO, or formic acid is reduced to CH_4, short alkanes, and/or solid organic material under hydrothermal conditions (Fig S9B). Experiments on abiotic hydrothermal reduction have shown CH_4 to be ^{13}C-depleted by as much as ~33‰, with

subsequent ethane and other short alkanes often less ^{13}C-depleted than the CH_4 (25) and references therein). Similarly, analyses of carbon compounds in volcanically-hosted waters, assuming that the reduced carbon gases formed from reduction of CO_2, show similar fractionation factors as the laboratory hydrothermal experiments (Fig. S9C). Carbon isotopic fractionation during microbial carbon fixation and microbial methanogenesis is summarized in Fig. S9D. Biological reduction of CO_2 to CH_4 can result in large fractionations under hydrogen limited co-culture conditions, but methanogen pure cultures demonstrate carbon isotopic fractionations similar to those of the hydrothermal experiments (Fig. S9D).

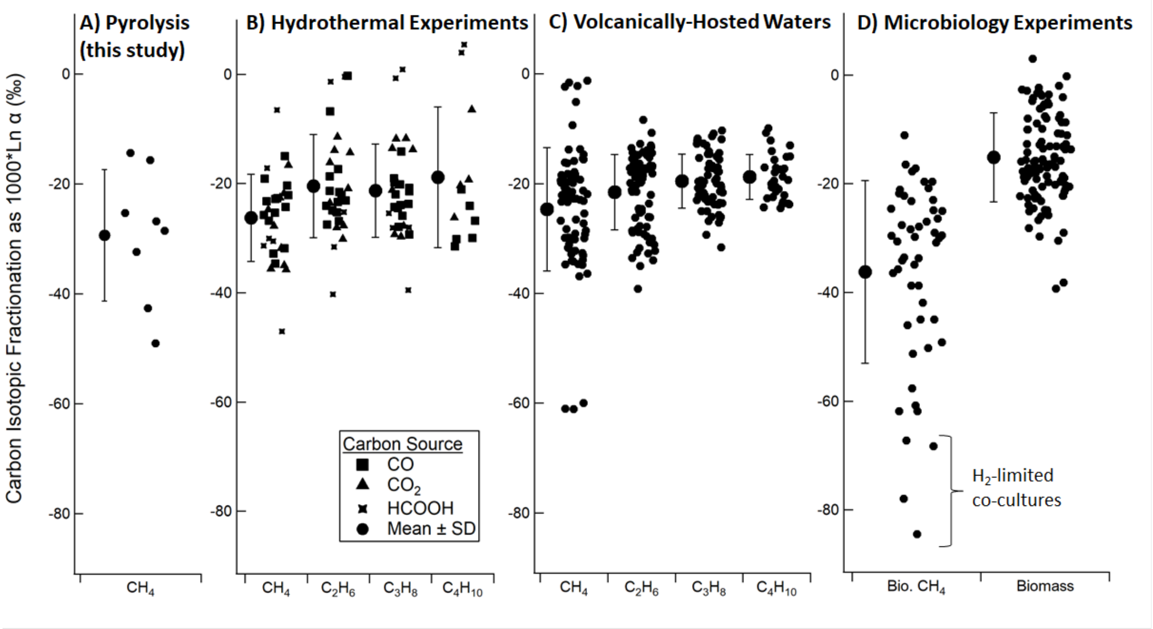

Figure S9. Carbon isotopic fractionation during (A) pyrolysis (this paper), (B) abiotic hydrothermal CO_2 reduction (25–32), (C) volcanic processes (33), and (D) microbial CO_2 fixation and methanogenesis (34–54). Small symbols show results from differing carbon substrates while large circles show the mean of all of the results ± 1 standard deviation.

SI References

1. J. Manne, T. Q. Bui, C. R. Webster, Determination of foreign broadening coefficients for Methane Lines Targeted by the Tunable Laser Spectrometer (TLS) on the Mars Curiosity Rover. *J. Quant. Spectrosc. Radiat. Transf.* **191**, 59–66 (2017).
2. H. B. Franz, *et al.*, Indigenous and exogenous organics and surface–atmosphere cycling inferred from carbon and oxygen isotopes at Gale crater. *Nat. Astron.* **4**, 526–532 (2020).
3. J. C. Stern, et al., Searching for Reduced Carbon on the Surface of Mars: The SAM Combustion Experiment in Eighth International Conference on Mars, (2014), p. 1419.
4. D. P. Glavin, *et al.*, Evidence for perchlorates and the origin of chlorinated

hydrocarbons detected by SAM at the Rocknest aeolian deposit in Gale Crater. *J. Geophys. Res. Planets* **118**, 1955–1973 (2013).
5. J. L. Eigenbrode, *et al.*, Organic matter preserved in 3-billion-year-old mudstones at Gale crater, Mars. *Science.* **360**, 1096–1101 (2018).
6. G. M. Wong, *et al.*, Detection of reduced sulfur on Vera Rubin ridge by quadratic discriminant analysis of volatiles observed during evolved gas analysis. *J. Geophys. Res. Planets* **125** (2020).
7. H. B. Franz, *et al.*, Large sulfur isotope fractionations in Martian sediments at Gale crater. *Nat. Geosci.* **10**, 658 (2017).
8. G. M. Wong, "Enhanced Evaluation of Martian Habitability in the Past and Present: The Search for Viable Electron Donors," Pennsylvania State University. (2020).
9. G. Wong, Processed data for figures and analysis in "Detection of reduced sulfur on Vera Rubin ridge by quadratic discriminant analysis of volatiles observed during evolved gas analysis" (2020) https:/doi.org/doi:10.7910/DVN/UOURYF.
10. F. Pedregosa, *et al.*, Scikit-learn: Machine Learning in Python. *J. Mach. Learn. Res.* **12**, 2825–2830 (2011).
11. A. Gilbert, K. Yamada, N. Yoshida, Exploration of intramolecular 13C isotope distribution in long chain n-alkanes (C11–C31) using isotopic 13C NMR. *Org. Geochem.* **62**, 56–61 (2013).
12. A. V. Milkov, G. Etiope, Revised genetic diagrams for natural gases based on a global dataset of >20,000 samples. *Org. Geochem.* **125**, 109–120 (2018).
13. R. Navarro-González, E. Vargas, J. de la Rosa, A. C. Raga, C. P. McKay, Reanalysis of the Viking results suggests perchlorate and organics at midlatitudes on Mars. *J. Geophys. Res. Planets* **115** (2010).
14. F. Keppler, *et al.*, Chloromethane release from carbonaceous meteorite affords new insight into Mars lander findings. *Sci. Rep.* **4**, 7010 (2014).
15. J. C. Stern, *et al.*, Evidence for indigenous nitrogen in sedimentary and aeolian deposits from the Curiosity rover investigations at Gale crater, Mars. *Proc. Natl. Acad. Sci.* **112**, 4245 LP – 4250 (2015).
16. C. Freissinet, *et al.*, Organic molecules in the Sheepbed Mudstone, Gale Crater, Mars. *J. Geophys. Res. Planets* **120**, 495–514 (2015).
17. J. Gropp, M. A. Iron, I. Halevy, Theoretical estimates of equilibrium carbon and hydrogen isotope effects in microbial methane production and anaerobic oxidation of methane. *Geochim. Cosmochim. Acta* **295**, 237–264 (2021).
18. R. D. Johnson III (ed.), NIST Computational Chemistry Comparison and Benchmark Database, NIST Standard Reference Database Number 101.
19. S.-L. Badea, *et al.*, Dehalogenation of α-hexachlorocyclohexane by iron sulfide nanoparticles: Study of reaction mechanism with stable carbon isotopes and pH variations. *Sci. Total Environ.* **801**, 149672 (2021).
20. H. C. Urey, The thermodynamic properties of isotopic substances. *J. Chem. Soc.*, 562–581 (1947).
21. J. Bigeleisen, M. G. Mayer, Calculation of Equilibrium Constants for Isotopic Exchange Reactions. *J. Chem. Phys.* **15**, 261–267 (1947).
22. G. M. Black, M. M. Law, The General Harmonic Force Field of Methyl Chloride. *J. Mol. Spectrosc.* **205**, 280–285 (2001).

23. D. L. Gray, A. G. Robiette, The anharmonic force field and equilibrium structure of methane. *Mol. Phys.* **37**, 1901–1920 (1979).
24. L. A. Leshin, *et al.*, Volatile, isotope, and organic analysis of martian fines with the Mars Curiosity rover. *Science.* **341** (2013).
25. T. M. McCollom, Laboratory Simulations of Abiotic Hydrocarbon Formation in Earth's Deep Subsurface. *Rev. Mineral. Geochemistry* **75**, 467–494 (2013).
26. Q. Fu, B. Sherwood Lollar, J. Horita, G. Lacrampe-Couloume, W. E. Seyfried, Abiotic formation of hydrocarbons under hydrothermal conditions: Constraints from chemical and isotope data. *Geochim. Cosmochim. Acta* **71**, 1982–1998 (2007).
27. G. Hu, Z. Ouyang, X. Wang, Q. Wen, Carbon isotopic fractionation in the process of Fischer-Tropsch reaction in primitive solar nebula. *Sci. China Ser. D Earth Sci.* **41**, 202–207 (1998).
28. T. M. McCollom, J. S. Seewald, Carbon isotope composition of organic compounds produced by abiotic synthesis under hydrothermal conditions. *Earth Planet. Sci. Lett.* **243**, 74–84 (2006).
29. T. M. McCollom, B. S. Lollar, G. Lacrampe-Couloume, J. S. Seewald, The influence of carbon source on abiotic organic synthesis and carbon isotope fractionation under hydrothermal conditions. *Geochim. Cosmochim. Acta* **74**, 2717–2740 (2010).
30. Y. A. Taran, G. A. Kliger, V. S. Sevastianov, Carbon isotope effects in the open-system Fischer–Tropsch synthesis. *Geochim. Cosmochim. Acta* **71**, 4474–4487 (2007).
31. Y. A. Taran, G. A. Kliger, E. Cienfuegos, A. N. Shuykin, Carbon and hydrogen isotopic compositions of products of open-system catalytic hydrogenation of CO2: Implications for abiogenic hydrocarbons in Earth's crust. *Geochim. Cosmochim. Acta* **74**, 6112–6125 (2010).
32. S. Zhang, J. Mi, K. He, Synthesis of hydrocarbon gases from four different carbon sources and hydrogen gas using a gold-tube system by Fischer–Tropsch method. *Chem. Geol.* **349-350**, 27–35 (2013).
33. J. Fiebig, *et al.*, Abiogenesis not required to explain the origin of volcanic-hydrothermal hydrocarbons. *Geochemical Perspect. Lett.* **11**, 23–27 (2019).
34. S. Hattori, *et al.*, Hydrogen and carbon isotope fractionation by thermophilic hydrogenotrophic methanogens from a deep aquifer under coculture with fermenters. *Geochem. J.* **46**, 193–200 (2012).
35. T. Okumura, *et al.*, Hydrogen and carbon isotope systematics in hydrogenotrophic methanogenesis under H2-limited and H2-enriched conditions: implications for the origin of methane and its isotopic diagnosis. *Prog. Earth Planet. Sci.* **3**, 14 (2016).
36. J. Penger, R. Conrad, M. Blaser, Stable carbon isotope fractionation by methylotrophic methanogenic archaea. *Appl. Environ. Microbiol.* **78**, 7596–7602 (2012).
37. A. L. Zerkle, C. H. House, S. L. Brantley, Biogeochemical signatures through time as inferred from whole microbial genomes. *Am. J. Sci.* **305**, 467 LP – 502 (2005).
38. A. Preuß, R. Schauder, G. Fuchs, W. Stichler, Carbon Isotope Fractionation by Autotrophic Bacteria with Three Different C02 Fixation Pathways. *Zeitschrift für Naturforsch. C* **44**, 397–402 (1989).

39. C. H. House, J. W. Schopf, K. O. Stetter, Carbon isotopic fractionation by Archaeans and other thermophilic prokaryotes. *Org. Geochem.* **34**, 345–356 (2003).
40. J. A. Calder, P. L. Parker, Geochemical implications of induced changes in C13 fractionation by blue-green algae. *Geochim. Cosmochim. Acta* **37**, 133–140 (1973).
41. J. W. Pardue, R. S. Scalan, C. Van Baalen, P. L. Parker, Maximum carbon isotope fractionation in photosynthesis by blue-green algae and a green alga. *Geochim. Cosmochim. Acta* **40**, 309–312 (1976).
42. H. Mizutani, E. Wada, Effect of high atmospheric CO2 concentration on δ13 of algae. *Orig. Life* **12**, 377–390 (1982).
43. S. Schouten, *et al.*, Stable Carbon Isotopic Fractionations Associated with Inorganic Carbon Fixation by Anaerobic Ammonium-Oxidizing Bacteria. *Appl. Environ. Microbiol.* **70**, 3785–3788 (2004).
44. M. T. J. van der Meer, S. Schouten, W. I. C. Rijpstra, G. Fuchs, J. S. Sinninghe Damsté, Stable carbon isotope fractionations of the hyperthermophilic crenarchaeon Metallosphaera sedula. *FEMS Microbiol. Lett.* **196**, 67–70 (2001).
45. M. T. J. van der Meer, et al., Biosynthetic Controls on the ^{13}C Contents of Organic Components in the Photoautotrophic Bacterium *Chloroflexus aurantiac*us. J. Biol. Chem. 276, 10971–10976 (2001).
46. M. T. Madigan, R. Takigiku, R. G. Lee, H. Gest, J. M. Hayes, Carbon isotope fractionation by thermophilic phototrophic sulfur bacteria: evidence for autotrophic growth in natural populations. *Appl. Environ. Microbiol.* **55**, 639–644 (1989).
47. K. L. Londry, D. J. Des Marais, Stable Carbon Isotope Fractionation by Sulfate-Reducing Bacteria. *Appl. Environ. Microbiol.* **69**, 2942–2949 (2003).
48. K. L. Londry, L. L. Jahnke, D. J. Des Marais, Stable Carbon Isotope Ratios of Lipid Biomarkers of Sulfate-Reducing Bacteria. *Appl. Environ. Microbiol.* **70**, 745–751 (2004).
49. S. S. Belyaev, *et al.*, Methanogenic Bacteria from the Bondyuzhskoe Oil Field: General Characterization and Analysis of Stable-Carbon Isotopic Fractionation. *Appl. Environ. Microbiol.* **45**, 691–697 (1983).
50. G. Fuchs, R. Thauer, H. Ziegler, W. Stichler, Carbon isotope fractionation by Methanobacterium thermoautotrophicum. *Arch. Microbiol.* **120**, 135–139 (1979).
51. L. Quandt, G. Gottschalk, H. Ziegler, W. Stichler, Isotope discrimination by photosynthetic bacteria. *FEMS Microbiol. Lett.* **1**, 125–128 (1977).
52. R. Sirevåg, B. B. Buchanan, J. A. Berry, J. H. Troughton, Mechanisms of CO2 fixation in bacterial photosynthesis studied by the carbon isotope fractionation technique. *Arch. Microbiol.* **112**, 35–38 (1977).
53. B. N. Popp, *et al.*, Effect of Phytoplankton Cell Geometry on Carbon Isotopic Fractionation. *Geochim. Cosmochim. Acta* **62**, 69–77 (1998).
54. L. L. Jahnke, *et al.*, Signature Lipids and Stable Carbon Isotope Analyses of Octopus Spring Hyperthermophilic Communities Compared with Those of Aquificales Representatives. *Appl. Environ. Microbiol.* **67**, 5179–5189 (2001).
